# Muscle metabolic resilience and enhanced exercise adaptation by Esr1-induced remodeling of mitochondrial cristae-nucleoid architecture in males

**DOI:** 10.1016/j.xcrm.2025.102116

**Published:** 2025-05-05

**Authors:** Zhenqi Zhou, Timothy M. Moore, Alexander R. Strumwasser, Vicent Ribas, Hirotaka Iwasaki, Noelle Morrow, Alice Ma, Peter H. Tran, Jonathan Wanagat, Thomas Q. de Aguiar Vallim, Bethan Clifford, Zhengyi Zhang, Tamer Sallam, Brian W. Parks, Karen Reue, Orian Shirihai, Rebeca Acin-Perez, Marco Morselli, Matteo Pellegrini, Sushil K. Mahata, Frode Norheim, Mingqi Zhou, Marcus M. Seldin, Aldons J. Lusis, Cathy C. Lee, Mark O. Goodarzi, Jerome I. Rotter, Joshua R. Hansen, Ben Drucker, Tyler J. Sagendorf, Joshua N. Adkins, James A. Sanford, Francesco J. DeMayo, Sylvia C. Hewitt, Kenneth S. Korach, Andrea L. Hevener

**Affiliations:** 1 David Geffen School of Medicine, Department of Medicine, Division of Endocrinology and Metabolism, University of California, Los Angeles, Los Angeles, CA 90095, USA; 2 Division of Geriatrics, David Geffen School of Medicine, Department of Medicine, University of California, Los Angeles, Los Angeles, CA 90095, USA; 3 Division of Cardiology, David Geffen School of Medicine, Department of Medicine, University of California, Los Angeles, Los Angeles, CA 90095, USA; 4 David Geffen School of Medicine, Department of Medicine, Department of Human Genetics, University of California, Los Angeles, Los Angeles, CA 90095, USA; 5 Department of Molecular, Cell and Developmental Biology and UCLA-DOE Institute for Genomics and Proteomics, University of California, Los Angeles, Los Angeles, CA 900095, USA; 6 Department of Medicine and VA, University of California, San Diego, La Jolla, CA 92037, USA; 7 University Department of Nutrition, Institute of Basic Medical Sciences, Faculty of Medicine, University of Oslo, 0316 Oslo, Norway; 8 Department of Biological Chemistry, University of California, Irvine, Irvine, CA 92697, USA; 9 Department of Medicine and VA, Greater Los Angeles Healthcare System GRECC, Los Angeles, CA 90073, USA; 10 Department of Medicine, Division of Endocrinology, Diabetes, and Metabolism, Cedars-Sinai Medical Center, Los Angeles, CA 90048, USA; 11 Institute for Translational Genomics and Population Sciences, The Lundquist Institute for Biomedical Innovation and Department of Pediatrics, Harbor-UCLA Medical Center, Torrance, CA, USA; 12 Chemical and Biological Signature Sciences Group, Pacific Northwest National Laboratory, Richland, WA 99354, USA; 13 Biological Sciences Division, Pacific Northwest National Laboratory, Richland, WA 99354, USA; 14 Reproductive Developmental Biology Laboratory, NIEHS, NIH, Research Triangle Park, NC 27709, USA; 15 Iris Cantor-UCLA Women’s Health Research Center, Los Angeles, CA 90095, USA

**Keywords:** estrogen action, insulin sensitivity, mitochondrial function, mtDNA replication, mitochondrial cristae architecture, oxidative metabolism, exercise adaptation

## Abstract

Reduced estrogen action is associated with obesity and insulin resistance. However, the cell and tissue-specific actions of estradiol in maintaining metabolic health remain inadequately understood, especially in men. We observed that skeletal muscle *ESR1*/*Esr1* (encodes estrogen receptor α [ERα]) is positively correlated with insulin sensitivity and metabolic health in humans and mice. Because skeletal muscle is a primary tissue involved in oxidative metabolism and insulin sensitivity, we generated muscle-selective *Esr1* loss- and gain-of-expression mouse models. We determined that *Esr1* links mitochondrial DNA replication and cristae-nucleoid architecture with metabolic function and insulin action in the skeletal muscle of male mice. Overexpression of human ERα in muscle protected male mice from diet-induced disruption of metabolic health and enhanced mitochondrial adaptation to exercise training intervention. Our findings indicate that muscle expression of *Esr1* is critical for the maintenance of mitochondrial function and metabolic health in males and that tissue-selective activation of ERα can be leveraged to combat metabolic-related diseases in both sexes.

## Highlights

• *ESR1* variants associate with insulin sensitivity in women and men
• Muscle ERα promotes mitochondrial fission dynamics and mtDNA replication
• Skeletal muscle-specific gain of *Esr1* expression protects against HFD-induced obesity
• Skeletal muscle *Esr1* drives mitochondrial adaptations to exercise training

## Graphical abstract

**Figure:**
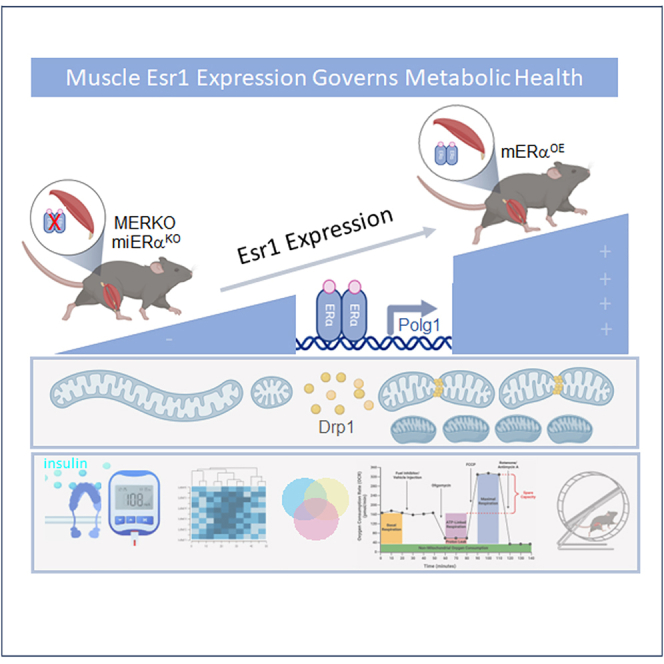

## Teaser

Zhou et al. identify *ESR1* variants that correlate with insulin sensitivity and metabolic health and recapitulate these relationships experimentally in mice by a muscle-selective alteration in *Esr1/ESR1* expression. Mitochondrial architecture and function as well as mitochondrial adaptation to physical activity are shown to be governed by *Esr1*/ERα.

## Introduction

Estrogen action is critical to metabolic health in both women and men. Recently, we determined that *ESR1* (the gene that encodes estrogen receptor α [ERα]) is more highly expressed than the androgen receptor across all metabolic tissues.^1^ Surprisingly, the number of cross-tissue transcripts significantly correlated with *ESR1* is 3-fold higher in males than females.^1^ Moreover, a rare inactivating mutation of *ESR1* (the gene encoding ERα) was shown to produce metabolic dysfunction in a middle-aged man,^2^^,^^3^^,^^4^^,^^5^ and male mice with a homozygous *Cyp19* (encodes aromatase) or *Esr1* null deletion mutation recapitulate many clinical features of metabolic syndrome.^6^^,^^7^ More recently, Finkelstein et al. showed that testosterone aromatization to estradiol is requisite for the beneficial effects of androgen replacement therapy on metabolism in men.^8^ In male mice, Garrett and colleagues showed that enhanced estrogen action by α-estradiol treatment improves substrate metabolism and extends median lifespan by 18%.^9^ Similarly, treatment of male mice with β-estradiol or ERα-selective agonist ameliorates high-fat diet (HFD)-induced obesity and insulin resistance.^10^ Although compelling pre-clinical and clinical evidence shows that estrogen action is indispensable for metabolic health in males, the cellular mechanisms underlying these effects remain inadequately understood. Considering that ERα is relatively well expressed in all metabolic tissues, the specific ERα-selective targets that drive metabolic health in both sexes require further interrogation.

To identify the tissue-specific actions of ERα governing metabolic health, we performed a tissue dissection approach in rodents using both conventional and conditional Lox-Cre strategies to manipulate *Esr1* expression in a cell-specific context.^3^^,^^11^^,^^12^^,^^13^^,^^14^^,^^15^ To date, phenotypic evaluation of animals with selective deletion of ERα from adipocytes, endothelial cells, hepatocytes, myeloid cells, and myocytes has been performed.^3^^,^^11^^,^^12^^,^^13^^,^^14^^,^^15^ However, deletion of ERα has predominantly been carried out during development, and therefore, compensatory mechanisms engaged during this life phase may obscure the role of ERα in regulating metabolism in adulthood. Moreover, conditional ERα overexpression models have yet to be examined.

Because we are interested in the molecular control of the metabolic and insulin-sensitizing actions of estradiol/ERα, we generated several unique mouse models to study the tissue-specific, temporal, and genomic actions of this receptor. We have previously shown in female mice that muscle-specific ERα knockout (MERKO) reduces oxidative metabolism and insulin sensitivity, and this mouse model recapitulated the obesity-insulin resistance phenotype of whole-body *Esr1*^−/−^ animals.^3^ Herein, we interrogated the relationship between *ESR1* and insulin sensitivity in men as well as a 100-strain panel of male inbred mice known as the UCLA Hybrid Mouse Diversity Panel (HMDP). We found that *Esr1* is highly correlated with insulin sensitivity as well as the expression of genes linked with mitochondrial fission and mtDNA replication, i.e., *Dnm1L* and *Polg1*, respectively. We used three genetic approaches including conventional and conditional gene deletion, as well as tissue-specific conditional overexpression to test the causal impact of *Esr1* expression on insulin sensitivity and metabolic health of male mice.

Deletion of *Esr1* from skeletal muscle produced insulin resistance, reduced myocellular oxidative metabolism, and disrupted metabolomic profiles. Insufficient nucleotide synthesis coupled with diminished ERα-driven expression of *Polg1* stalled mtDNA replication and produced morphological changes in mitochondrial nucleoids, cristae composition, as well as inner and outer mitochondrial membrane architecture. In contrast to the muscle ERα deletion mouse model, mERα^OE^ enhanced skeletal muscle insulin sensitivity and protected against diet-induced insulin resistance by enhanced mitochondrial fission dynamics, cristae remodeling, and oxidative capacity. These cellular adaptations likely underpinned improvements in exercise training-induced adaptations in mERα^OE^. Collectively, our findings suggest that skeletal muscle ERα is critical for the maintenance of mitochondrial function and that muscle *ESR1* may be an effective target to combat diseases associated with metabolic dysfunction.

## Results

### *ESR1* and insulin sensitivity in human subjects and inbred mouse panel

Single-nucleotide polymorphisms (SNPs) in *ESR1* have been associated with a variety of disease-related traits including adiposity,^16^ mammographic density,^17^ and bone mineral density.^18^ Furthermore, precedence for a male-specific association between ERα gene variation and measures of adiposity and cardiovascular disease risk has also been observed.^5^^,^^19^ Analysis of genome-wide association studies^20^ revealed two independent genetic variants in *ESR1* (rs2982712 and rs9479103; Figures S1A–S1C). These variants were identified at opposite ends of the gene and are significantly correlated with insulin sensitivity, independent of age and sex (glucose disposal rate [GDR] as assessed by the gold-standard hyperinsulinemic-euglycemic clamp^1^^,^^11^) (Figures S1A–S1C). Three additional SNPs were associated with diabetes risk in the Women’s Health Initiative WHI SHARe data (https://www.whi.org/; WHI Harmonized and Imputed GWAS Data A sub-study of Women’s Health Initiative dbGaP Study Accession: phs000746.v3.p3; Figure S1C). The relationships between muscle *ESR1* expression and indices of metabolic health appear similar between mice and humans, as well as between the sexes.^3^ Interrogation of Stockholm-Tartu Atherosclerosis Reverse Networks Engineering Task,^21^ which includes RNA sequencing of 9 tissues from 850 subjects, 600 with coronary artery disease (CAD; 70% male) and 250 without CAD (55% males), indicates that skeletal muscle *ESR1* expression is inversely correlated with CAD risk (−0.14475, *p* value = 0.00085399). Despite significantly higher basal *ESR1* expression levels in liver compared with skeletal muscle, no correlation between *ESR1* expression in liver and CAD risk was observed (*p* value = 0.38893).^21^

**Figure 1:**
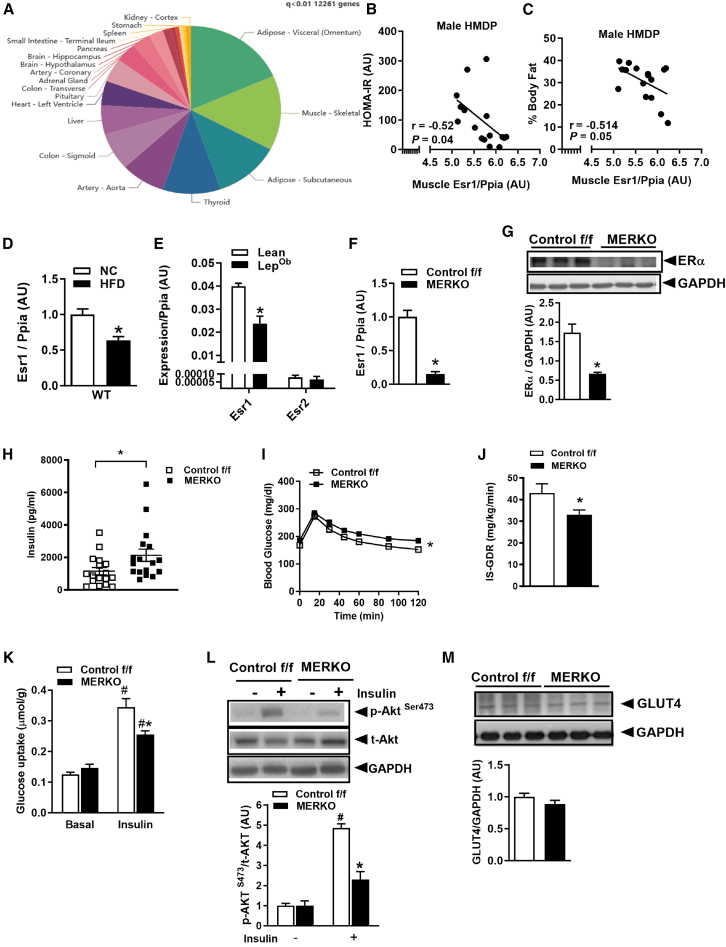
Muscle ERα controls insulin sensitivity and oxidative metabolism (A) *ESR1* in muscle from men shows a high number of gene-gene correlations and limited overlap with other metabolic tissues outside of adipose (GTEx *n* = 210 males, age 20–70 years). (B and C) Muscle *Esr1* is inversely correlated with the HOMA-IR insulin resistance index and adiposity (% fat) in a 16-strain panel of inbred mice (*n* = 4 mice per strain for individual closed circles). (D and E) Muscle *Esr1* expression is reduced in male high-fat diet-fed and *Lep*^*Ob*^ mice vs. lean controls (*n* = 6/group). (F and G) *Esr1* mRNA and protein in muscle from control f/f and MERKO male mice (representative immunoblot, *n* = 6/genotype). (H–K) (H) Fasting plasma insulin in MERKO vs. control f/f (*n* = 17 mice/genotype). (I) Glucose tolerance test in control f/f and MERKO male mice (*n* = 6–8/group). Skeletal muscle insulin sensitivity assessed by (J) hyperinsulinemic-euglycemic clamp (IS-GDR, insulin-stimulated glucose disposal rate; *n* = 6–8/genotype) and (K) *ex vivo* soleus muscle glucose uptake (*n* = 5–6 mice/genotype) in control f/f and MERKO male mice. (L) Representative immunoblots of insulin-stimulated *p*-Akt in basal 6-h-fasted quadriceps muscle (*n* = 6/genotype). (M) GLUT4 protein levels in basal 6-h-fasted quadriceps muscle (*n* = 5–6/genotype). Densitometric analyses expressed in arbitrary units (AU). All values expressed as means ± SEM, ∗*p* < 0.05 between genotype comparison, # within genotype between condition comparison. Significance detected by Student’s t test and repeated measures ANOVA where appropriate.

Moreover, we interrogated human transcriptomics data from the Genotype-Tissue Expression Program (GTEx) and identified a high number of transcripts significantly correlated with *ESR1* in skeletal muscle (12,261, *q* < 0.01), and many of these gene-gene correlations were overlapping in visceral and subcutaneous adipose tissue (Figure 1A). The top 30 gene correlations are provided (Figure S2A; *p* < 0.001), and gene set enrichment analysis revealed top pathways including electron transport chain, mitochondrial respirasome, mitochondrial ATP synthesis, extracellular matrix, sarcomere, and myofibril, positively associating with *ESR1* (Figure S2B; left panel). Pathways negatively associated with *ESR1* included small subunit processome, preribosome, ribonucleoprotein complex assembly, and RNA splicing (Figure S2B; right panel). Collectively, these data confirm that there are a high number of *ESR1*-gene correlations in skeletal muscle and that muscle *ESR1* expression is relevant to the regulation of nuclear-encoded mitochondrial-related transcripts as well as cardiometabolic disease risk in men.

Next, we utilized a large panel of inbred and recombinant inbred mice known as the UCLA HMDP to interrogate sex differences in muscle transcripts associated with *Esr1* (Figures S3A–S3D). Computational analyses of RNA sequencing performed on muscle (gastrocnemius) from male and female mice of the HMDP showed that 10,517 transcripts were significantly associated with *Esr1* (false discovery rate *p* < 10^−7^) in females, whereas 2,661 transcripts in total were significantly associated with *Esr1* in male animals. Of interest, ∼99% of transcripts significantly correlated with *Esr1* in males, overlapped with females (Figure S3B; Venn diagram). Functional annotation clustering of these transcripts showed mitochondrion as the top category of enrichment for both males and females (Figure S3C; left lower panel). Overlapping processes divergent for Esr1 correlation, suggesting potential differential regulation between the sexes, included response to hypoxia (Figure S3D, right lower panel). These findings show that *Esr1* expression strongly associates with a high number of transcripts in mouse muscle and that the genes correlated with *Esr1* are largely overlapping in males and females, with the mitochondrion as an important regulatory node of estrogen action. With respect to gene-trait assessment, expression of *Esr1* in muscle is inversely correlated with the Homeostatic Model Assessment for Insulin Resistance (HOMA-IR) and body fat percent (Figures 1B and 1C). These findings substantiate the notion that skeletal muscle expression of *ESR1* may be an important variable in understanding metabolic disease risk in both sexes.

### Impaired glucose homeostasis and insulin action in male MERKO mice

Although strong relationships between *ESR1* expression and metabolic health indices have been shown in human subjects, the causal mechanisms and tissues of *ESR1* action underlying the maintenance of oxidative metabolism and insulin sensitivity remain incompletely understood. To interrogate causality, we generated mouse models with deletion or overexpression of *Esr1* in skeletal muscle during development or adulthood.

Although ERα is often more highly expressed in metabolic tissues of females compared with males, it was shown that whole-body homozygous null deletion of *Esr1* in male mice produced metabolic dysfunction similar to that reported for females.^3^ Moreover, *Esr1* expression is reduced in muscle from male high-fat-fed (wild type) and genetically obese (*Lep*^*Ob*^) animals (Figures 1D and 1E). No difference in *Esr2* expression between the respective groups was noted, and *Esr2* was expressed at appreciably lower levels in skeletal muscle compared with *Esr1* (Figure 1E). These data confirm our published findings in females showing that diet- and genetic-induced metabolic dysfunction is associated with reduced *Esr1* expression levels.^3^

Because *Esr1* shows strong heritability (SNP heritability h^2^ = 0.62 mice) but wide variability in muscle expression among the mouse strains, we studied animals with a muscle-specific deletion of *Esr1* induced by muscle creatine kinase Cre recombinase (MERKO). Both *Esr1* transcript (Figure 1F) and ERα protein (Figure 1G) levels were reduced in muscle from male MERKO mice, but not heart or liver (evidence of tissue specificity Figure S4A). Similar to female MERKO animals, male mice showed an increase in body weight and white adipose tissue mass with aging (Figures S4B and S4C). Increased body weight and adiposity were recapitulated in the muscle-specific inducible *Esr1* knockout (KO) model (miERα^KO^) in the context of HFD feeding after only 8 weeks of gene deletion (Figures S4D and S4E).

In parallel to the increase in adiposity, there was an increase in circulating leptin (↑50%; *p* = 0.04) and insulin (↑85%; *p* = 0.03; Figure 1H) in plasma of male MERKO mice compared with control f/f (NC fed). Male MERKO mice showed impaired glucose tolerance (AUC, area under curve; *p* = 0.01) compared with control f/f (Figure 1I), similar to females.^3^ To determine the tissues contributing to this impairment in glucose homeostasis, we performed hyperinsulinemic-euglycemic clamp studies. The insulin-stimulated GDR, primarily reflecting skeletal muscle insulin sensitivity, was reduced by 23% in MERKO mice under NC-fed conditions (Figure 1J). The insulin resistance phenotype was also observed in the muscle-specific inducible model after 8 weeks of gene deletion (Figure S4F). Consistent with findings from glucose clamps, in a separate cohort of animals, we observed a 42% (*p* = 0.01) reduction in *ex vivo* insulin-stimulated 2-deoxyglucose uptake into soleus muscle (Figure 1K), which was paralleled by a marked reduction in insulin-stimulated phosphorylation of Akt in MERKO muscle compared with control f/f (Figure 1L). The insulin resistance phenotype was explained by defects in insulin signaling and not by total abundance of the insulin-responsive glucose transporter, GLUT4 (Figure 1M). Collectively, our findings confirm that a loss of ERα promotes skeletal muscle insulin resistance in both female and male mice.

### Lipids and oxidative metabolism in MERKO mice

**Figure 2:**
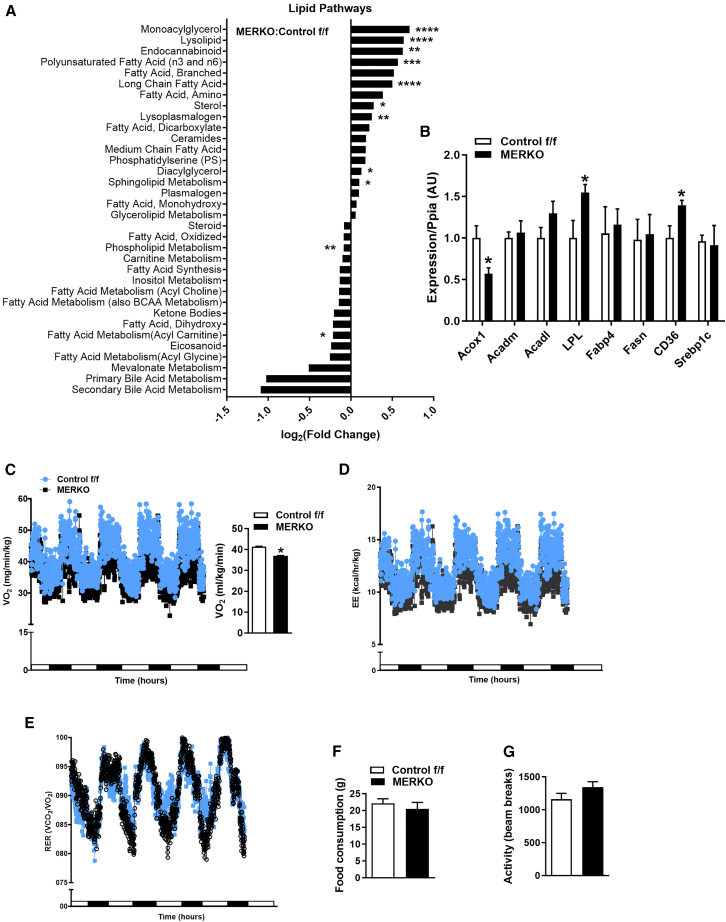
Muscle-specific ERα deletion reduces basal energy expenditure promoting muscle lipid accumulation (A) Lipidomic analyses of muscle from control f/f and MERKO mice, *n* = 6/genotype. (B) Quantitative reverse-transcription PCR (RT-PCR) analysis of quadriceps muscle transcripts reflecting lipid metabolism (*n* = 6/genotype). (C–G) (C and D) Metabolic caging studies using indirect calorimetry to determine (C) VO_2_, (D) energy expenditure, (E) respiratory exchange ratio (RER), (F) food consumption, and (G) ambulatory activity (*n* = 6 per genotype). All values are expressed as means ± SEM detected by Student’s t test and ANCOVA where appropriate. ∗*p* < 0.05 between genotype difference.

Impaired skeletal muscle insulin action in MERKO muscle coincided with accumulation of a variety of lipid species compared with control f/f (Figure 2A). A robust increase in monoacylglycerol, proinflammatory phospholysolipids, long-chain fatty acids, diacylglycerol, and sphingolipids was observed in MERKO muscle (Figure 2A). Elevated muscle lipids were associated with increased expression of lipid regulatory genes including *LPL* and *CD36* (fatty acid translocase) involved in the hydrolysis of triglycerides from lipoproteins as well as fatty acid transport into muscle, respectively (Figure 2B). In contrast, *Acox1* (acyl-CoA oxidase 1) expression was reduced by ∼50% in MERKO mouse muscle (Figure 2B). *Acox1* encodes the first and rate-limiting enzyme of the very-long-chain fatty acid beta-oxidation pathway in peroxisomes, which catalyzes the desaturation of acyl-CoAs to 2-trans-enoyl-CoAs. A reduction in *Acox1* expression is internally consistent with increased abundance of long-chain fatty acids in muscle of male MERKO mice, and this finding was recently recapitulated in mice with a muscle-selective deletion of the mitochondrial fission regulator dynamin-related protein (DRP)-1.^22^ *In vivo* oxygen consumption, determined by indirect calorimetry, and energy expenditure were reduced in MERKO mice compared with control f/f (Figures 2C and 2D), whereas respiratory exchange ratio was elevated in MERKO, suggesting a greater reliance on carbohydrate as a fuel source (Figure 2E). No differences in food consumption and ambulatory movement were observed between the genotypes (Figures 2F and 2G). These findings suggest that muscle ERα drives oxidative metabolism and that reduced ambulatory movement observed in *Esr1* null mice^6^ is a phenotype governed by extramyocellular mechanisms.

### ERα controls mitochondrial form and function

**Figure 3:**
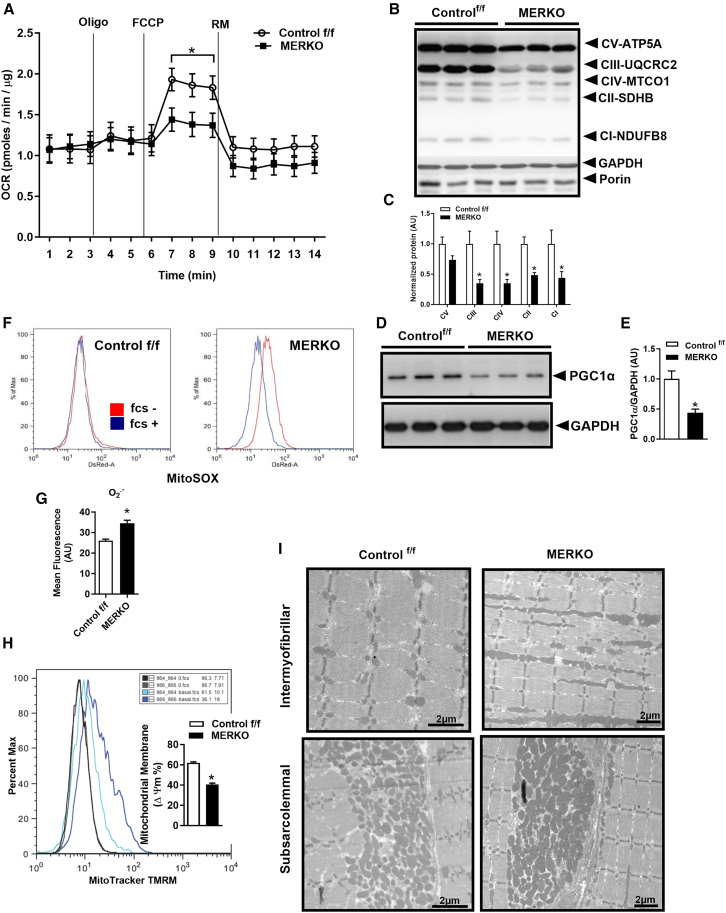
ERα deletion alters mitochondrial morphology and respiration (A) Real-time respirometry was performed on frozen quadriceps muscle homogenates from control f/f and MERKO mice (*n* = 6 mice/genotype). (B and C) Representative immunoblots and corresponding densitometry of representative subunits of the mitochondrial electron transport complexes in muscle from control f/f and MERKO (*n* = 6/genotype). (D and E) Representative immunoblot and densitometry of muscle PGC1α from control f/f and MERKO mice (*n* = 6/genotype). (F–H) (F and G) Primary muscle cells were stained with Mitosox reflecting superoxide and (H) TMRM reflecting mitochondrial membrane potential and analyzed by flow cytometry (*n* = 3 studies performed in duplicate). (I) Transmission electron microscopy images showing elongated and hyperfused mitochondria in MERKO (right) vs. control f/f (left). Scale bars: 2 μm. All values are expressed as means ± SEM detected by Student’s t test and repeated measures ANOVA where appropriate. ∗*p* < 0.05 between genotype difference.

Because we observed a marked accumulation of fat in skeletal muscle of MERKO mice, we hypothesized that ERα may be important in the regulation of oxidative metabolism by mitochondria. To determine the impact of ERα on mitochondrial function, we performed real-time respirometry studies in primary myotubes from both genotypes of mice and from C2C12 myotubes with lentiviral-mediated Esr1 knockdown (KD). We observed reduced carbonyl cyanide-p-trifluoromethoxyphenylhydrazone (FCCP)-stimulated maximal respiration in both ERα mutant cell preparations tested as well as frozen muscle homogenates from MERKO vs. control f/f (Figure 3A). In support of ERα controlling oxidative phosphorylation, we observed a >50% reduction in representative subunits of complex I–IV in muscle from male MERKO mice (Figures 3B and 3C), and although we observed no difference in transcript abundance of markers of mitochondrial biogenesis, we did observe a marked reduction in protein abundance of PGC1α vs. control f/f (Figures 3D and 3E). Superoxide production as assessed by flow cytometry analysis using Mitosox showed a 30% elevation in Esr1-KD over control (Scr) (*p* = 0.02; Figures 3F and 3G). Moreover, membrane potential was reduced by 33% (*p* = 0.002) in the context of ERα deficiency vs. control (Figure 3H).

Since mitochondrial function was disrupted in MERKO muscle, we determined whether morphological changes in mitochondrial architecture were correlated with functional alterations in metabolism. Electron micrographs of soleus muscle from male MERKO mice showed enlarged, tubulated, and hyper-fused mitochondria compared with control (Figure 3I). This observation is reminiscent of the mitochondrial architecture in aged muscle.^23^^,^^24^^,^^25^^,^^26^^,^^27^ Mitochondrial hyperfusion is often reflective of organelle stress and could be indicative of a compensatory response to stabilize the genome, dilute damaged mitochondrial content across the mitochondrial network, and prevent autophagic degradation of mitochondria by lysosomes.^28^^,^^29^^,^^30^^,^^31^^,^^32^

### ERα regulates mitochondrial fission-fusion dynamics signaling

**Figure 4:**
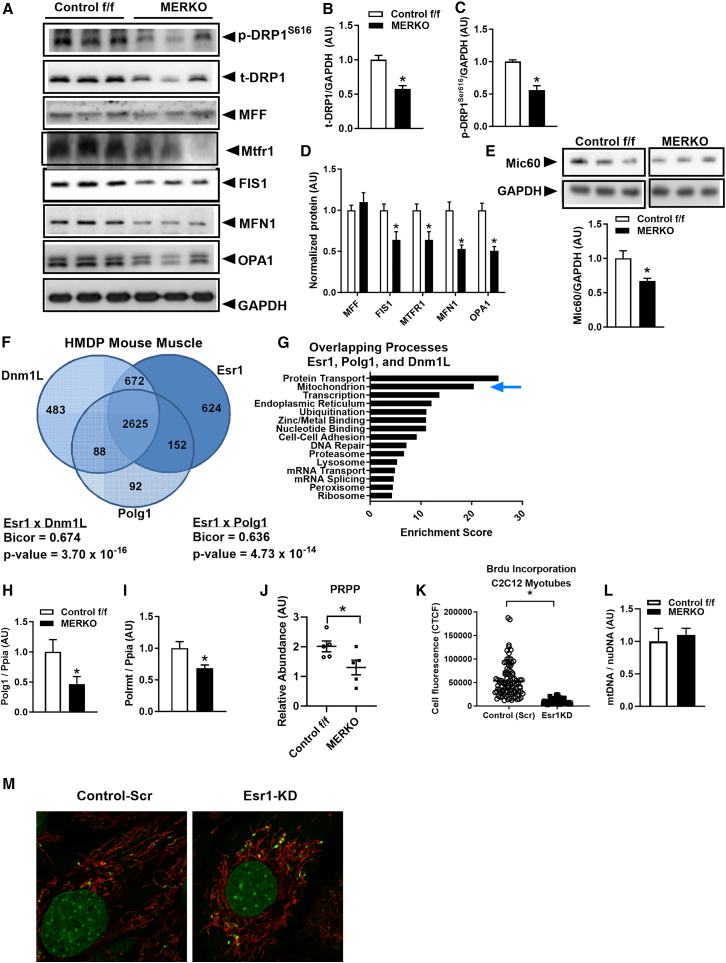
Muscle ERα deletion alters mitochondrial fission-fusion signaling and mtDNA replication (A–E) Representative immunoblots and corresponding densitometry of mitochondrial fission-fusion proteins and cristae junction-related proteins from control f/f vs. MERKO mouse quadriceps muscle (*n* = 6/genotype). (F) RNA sequencing of gastrocnemius muscle from the 100-strain UCLA Hybrid Mouse Diversity Panel (HMDP) with Venn diagram showing the gene overlap between *Esr1*, *Dnm1L*, and *Polg1* (*n* = 4 mice/strain). (G) Enrichment score analysis of muscle RNA sequencing from HMDP mice shows that *Esr1*, *Polg1*, and *Dnm1L* associate with transcripts involved in protein transport and mitochondria. (H and I) (H) *Polg1* and (I) *Polrmt* expression are markedly reduced in muscle from NC-fed MERKO vs. control f/f mice (*n* = 6/genotype). (J) Reduced PRPP in MERKO vs. control f/f detected by metabolomics analysis (*n* = 5–6/genotype). (K) Reduced Brdu incorporation into newly synthesized mtDNA from C2C12 myotubes with Esr1-KD vs. scrambled control (Scr) (*n* = 3 in triplicate). (L and M) (L) Mitochondrial DNA copy number in muscle is identical between MERKO and control f/f mice (*n* = 6/genotype). (M) Confocal microscopy images show enlarged mtDNA-containing nucleoids (stained with Picogreen) in cells lacking Esr1, along with reticular-networked mitochondria (stained with Mitotracker red). All values are expressed as means ± SEM detected by Student’s t test. ∗*p* < 0.05 between genotype difference.

Consistent with a hyperfused mitochondrial phenotype, proteins involved in fission-fusion dynamics were dysregulated in male MERKO muscle. Most notably, DRP1, critical for mitochondrial fission, was reduced by nearly 50% in muscle lacking *Esr1* (Figures 4A and 4B). DRP1 phosphorylation^S616^ was also reduced, reflective of mitochondrial fission incompetence (Figures 4A and 4C). The mitochondrial outer membrane receptor for DRP1-induced fission, FIS1, and mitochondrial fission-related factor were similarly reduced in MERKO muscle compared with control f/f (Figures 4A and 4D), and collectively, these data are congruous with the elongated hyperfused mitochondrial phenotype. In contrast to female MERKO mouse muscle in which mitochondrial fusion was accompanied by an induction of fusion regulatory protein abundance, the expression of mitofusin (MFN)1 and optic atrophy (OPA) 1 proteins was reduced by almost 50% in the muscle of male MERKO mice compared with control (Figures 4A and 4D). OPA1 is a multi-functional protein with actions beyond inner membrane fusion including the regulation of cristae morphology and cristae junction conformation.^33^ Alterations in OPA1 are also consistent with reduced protein expression of MICOS protein Mic60 (also known as mitofilin),^34^ which controls cristae architecture and inner-outer membrane contacts (Figure 4E). Although we observed a marked reduction of proteins controlling fission-fusion dynamics, we observed no change in expression of corresponding transcripts (Figure S4G). Beyond a role for ERα in the regulation of transcription, poor RNA:protein concordance is consistent with ERα controlling the translation of mitochondrial-related proteins and or the activity of specific proteases that modulate mitochondrial protein turnover.

### ERα regulates mtDNA replication and translation

Although we observed no alteration in *Dnm1L* expression in male MERKO muscle, our findings in the 100-strain mouse panel show a strong positive relationship between *Esr1*, *Dnm1L*, and *Polg1* (Figure 4F) and a remarkable overlap in transcripts associated with each of these genes. Genome-wide association studies suggest that a primary action of *Esr1* includes the regulation of the mitochondrial life cycle, specifically mtDNA replication and dynamics. Remarkably, we found a statistically significant overlap between *Esr1* vs. *Dnm1L*-correlated genes, Bicor = 0.674 (*p* = 3.7 × 10^−16^), and *Esr1* vs. *Polg1*, Bicor = 0.636 (*p* = 4.73 × 10^−14^) (Figure 4F). The shared metabolic processes enriched for all three transcripts included mitochondrion, protein transport, transcription, and nucleotide binding (Figure 4G).

Because *Polg1*, the gene that encodes the catalytic subunit of the only known mammalian mtDNA polymerase PolG,^3^ is strongly correlated with *Esr1* expression in the muscle of both female and male mice, we interrogated the mechanistic role of ERα in the regulation of *Polg1* expression and mtDNA copy number (CN). *Polg1* expression levels were markedly reduced in an age- and dose-dependent manner with *Polg1* transcript reduced by ∼50%–75% in MERKO muscle as early as 2 months of age, with similar reductions in transcript between muscle-specific *Esr1* heterozygous and homozygous deletion mice vs. f/f controls (Figures S4H and 4H). Moreover, PolG protein abundance was reduced in miERα^KO^ following short-term reduction of *Esr1* (Figure S4I).

In contrast to *Polg1*, no differences in the expression of the mtDNA polymerase accessory subunit *Polg2* (encodes a 55 kDa accessory subunit protein) or the G elongation mitochondrial factor 2, *Gfm2*, involved in translation were detected between the genotypes (Figure S4J). However, *Polrmt*, responsible for mitochondrial gene expression as well as for providing RNA primers for initiation of replication of the mitochondrial genome, was markedly reduced in the muscle of male MERKO mice compared with control f/f (Figure 4I). This observation is unique to males as we detected no difference in *Polrmt* expression in female MERKO vs. control f/f.^3^ Thus, it is presumed that reduced expression of both *Polrmt* and *Polg1* stalled mtDNA replication.^3^ This notion is supported by reduced levels of 5-phosphoribosyl diphosphate (Figure 4J), a pentose phosphate intermediate in purine and pyrimidine nucleotide synthesis, as well as pentose species ribonate (0.67 MERKO:control f/f; *p* < 0.05) and arabitol/xylitol (0.79 MERKO:control f/f; *p* < 0.05) (Table S1).

Consistent with reduced *Polg1* and *Polrmt*, we observed reduced Brdu incorporation into newly synthesized mtDNA (Figure 4K), suggesting a stall in nucleotide incorporation into mtDNA. So, although mtDNA CN was identical between the genotypes (Figure 4L) phenocopying published findings for female mice, our work indicates that the reduction in mtDNA replication is matched by a coordinated reduction in mitophagy; thus, maintenance of the mtDNA CN is achieved at the expense of mitochondrial health. mtDNA replicative stress is consistent with our observation by fluorescence imaging that mitochondrial nucleoids, the discrete protein packaging of the mitochondrial genome, are enlarged and aggregated in MERKO myotubes vs. control f/f (Figure 4M; mtDNA green puncta, mitochondrial network in red). Mitochondrial stress is further supported by proteomic analyses showing attempted, but ineffective, compensation by the mitochondrial ribosomal compartment in MERKO vs. control (Figure S5).

Moreover, insufficient supply of phosphoribosyl pyrophosphate (PRPP; ↓ 0.65 MERKO:control f/f, Figure 4J) is another contributor to the reduced nucleotide synthesis, as PRPP is required for both *de novo* and salvage pathways. With respect to pyrimidine metabolism, we observed a decrease in orotate and uracil as well as NAD (↓ 0.51 MERKO:control f/f, *p* < 0.05). In addition, changes in the following metabolites, AMP (↓ 0.65 MERKO:control f/f, *p* < 0.05), guanine (↑ 1.65 MERKO:control f/f, *p* < 0.05), and purine catabolites (↓ allantoin 0.78, urate ↓ 0.28, xanthine ↓ 0.81 MERKO:control f/f, *p* < 0.05), support alterations in nucleotide synthesis and/or differences in repair processes (Table S1). Changes in redox homeostasis associated with ERα deletion were also observed. A significant decrease in reduced glutathione (↓ 0.37 MERKO:control f/f, *p* < 0.05) and increased methionine sulfoxide (↑ 1.65 MERKO:control f/f, *p* < 0.05) and N-acetylmethionine sulfoxide (↑ 1.55 MERKO:control f/f, *p* < 0.05) in male and female MERKO muscle vs. control f/f reflects an oxidizing environment consistent with elevated mitochondrial ROS production in MERKO muscle and *Esr1*-deficient myotubes compared with control ERα-replete muscle (Table S1). Thus, these findings indicate that ERα may directly control the transcriptional machinery for mtDNA replication, as well as nucleotide *de novo* and salvage pathways.

#### Drp1-targeted degradation by Parkin is heightened in muscle devoid of ERα

Based upon observations linking mtDNA replication with mitochondrial fission-fusion dynamics,^35^ we hypothesized that a reduction in *Polg1* expression and a stall in mtDNA replication likely promote feedback inhibition of all aspects of the mitochondrial life cycle. Since mtDNA CN was maintained in MERKO mice similar to controls despite a reduction in mtDNA replication, this suggests that the rate of mitophagic turnover must be coordinately reduced. To interrogate the impact of ERα expression on mitophagy, we determined the expression of known mitophagy regulators and flux in the macroautophagic pathway. First, we observed that both *Park 6* (PINK1) and *Park 2* (Parkin) expression levels were reduced in male MERKO muscle vs. control f/f (Figure S6A). This is in contrast to observations for female animals where no difference in transcript abundance was detected between genotypes.^3^ Additionally, in sharp contrast to our observations for female MERKO mice,^3^ we detected a marked increase in protein abundance of both full (63 kDa) and cleaved (52 kDa) PINK1, as well as Parkin total protein (Figures S6B–S6F). Parkin protein phosphorylation (Ser65, the putative activation site) was reduced in MERKO muscle vs. control f/f (Figures S6B and S6D).

Since cytosolic cleaved PINK1 is known to inhibit Parkin translocation, next we tested the distribution of Parkin in the cell by quantifying the cytosolic and outer mitochondrial membrane fractions. We observed less Parkin protein in the mitochondrial fraction and increased abundance in the cytosol for MERKO muscle compared with control f/f (Figures S6E and S6F).

Because we detected a reduced abundance of Drp1 in both the total lysate and mitochondrial fractions from MERKO mice and C2C12 cells with Esr1-KD vs. control, we determined whether Parkin binds Drp1 in the cytosol and is involved in Drp1 depletion in the context of *Esr1* deletion. We expressed Parkin-flag in C2C12 cells to determine a time and dose-dependent effect on Drp1 protein expression in total cell lysate. Indeed, we observed a concentration-dependent reduction in Drp1 with increasing Parkin, as well as a reduction of Drp1 over time in the context of fixed Parkin overexpression (Figures S6G and S6H). Indeed, Drp1 immunoprecipitation studies showed a 2-fold increase in Parkin protein binding, as well as a marked increase in Drp1-associated ubiquitin in C2C12 cells with Esr1-KD (Figure S6I).

We treated C2C12 cells with inhibitors of the proteasome (MG132), lysosome (BafA1), and protein synthesis (CHX, cyclohexamide) to determine the primary pathway for Drp1 turnover in the context of *Esr1* deletion (Figures S6J and S6K). Inhibition of calpain proteases led to a 2-fold increase in Drp1 protein levels, suggesting that the absence of ERα from muscle cells increases Parkin-directed turnover of Drp1 protein by ubiquitin-mediated proteasomal degradation.

Since ERα controls Drp1 and Parkin action indirectly, not by direct DNA binding, we determined if specific reduction of *Polg1*, a *bona fide* ERα target gene, impacted Drp1 protein expression. Indeed, deletion of *Polg1* in C2C12 cells reduced Drp1 total protein similar to our previous observation for *Esr1-*KD (Figures S6L and S6M). These data reveal a novel regulatory axis between ERα-Polg1-Parkin-Drp1 to coordinate mtDNA replication with mitochondrial fission and organelle quality control.

### Muscle-specific Esr1 overexpression remodels mitochondrial cristae architecture and drives mtDNA replication to enhance oxidative metabolism

**Figure 5:**
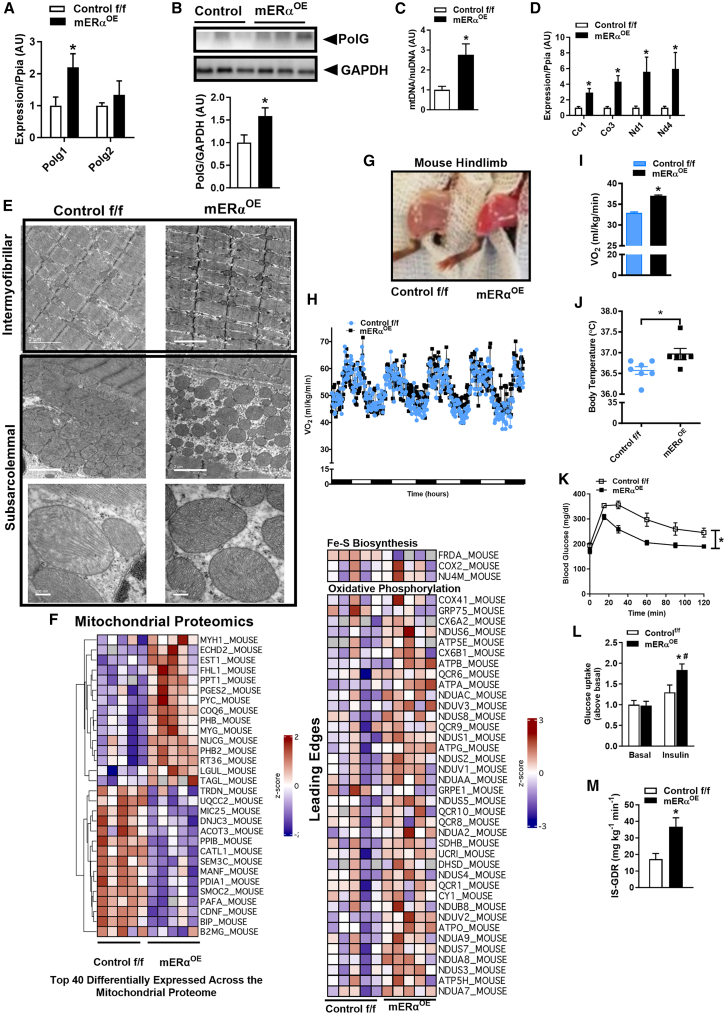
Muscle-specific *Esr1* overexpression increases mitochondrial function and metabolic health (A and B) *Polg1* transcript and PolG protein expression (representative immunoblot and densitometry) are markedly increased in quadriceps muscle from mERα^OE^ vs. control f/f (*n* = 6 mice/genotype). (C and D) (C) Muscle mitochondrial DNA copy number and (D) expression of mitochondrial-encoded transcripts are increased in mERα^OE^ vs. control f/f (*n* = 6 mice/genotype). (E) Transmission electron microscopy images of muscle from mERα^OE^ (right) vs. control f/f (left) showing that *Esr1* overexpression promotes a more spherical (lower) electron-dense organelle with increased cristae volume (*n* = 4 mice/genotype). Scale bars: 2 μm for top 4 panels and 200 nm for lower 2 panels of higher magnification images. (F) Heatmap showing top 40 differentially expressed mitochondrial proteins between mERα^OE^ and control f/f (*n* = 5 per genotype; quadriceps muscle). (G–J) (G) Hindlimb muscles from mERα^OE^ show a deeper red color compared with control f/f (*n* = 6 mice/genotype). Metabolic caging studies show that mERα^OE^ mice have an (H and I) increased VO_2_ and (J) body temperature compared with control f/f (*n* = 6–7 mice/genotype). (K–M) Muscle-specific *Esr1* overexpression protects male mice from HFD-induced (K) glucose intolerance (AUC, area under the curve) and (L and M) muscle insulin resistance determined by *ex vivo* muscle 2-deoxyglucose uptake and hyperinsulinemic-euglycemic clamps, respectively (*n* = 6 mice/genotype). All values are expressed as means ± SEM detected by Student’s t test. ∗*p* < 0.05 between genotype difference.

Because we have previously shown that ERα is induced by exercise training and associated with improvements in metabolic health, we generated a mouse model in which we conditionally overexpressed human *ESR1*/ERα in skeletal muscle (mERα^OE^) of adult animals (mERα^OE^ model construct; Figure S7A). The increase in ERα protein in male mERα^OE^ mice was paralleled by a muscle-selective induction of *Polg1/*PolG (Figures 5A and 5B), the protein known to govern mtDNA replication. Increased *Polg1* expression was associated with a ∼3-fold increase (*p* = 0.001) in mtDNA CN (Figure 5C). MtCOI, MtCO3 (mitochondrial-encoded cytochrome *c* oxidase), ND1, and ND4 expression was also increased in muscle from mERα^OE^ vs. control f/f (Figure 5D). Although we observed minimal alteration in the mitochondrial outer membrane morphology between the genotypes (Figure 5E), the cristae were more densely packed in *ESR1*-overexpressing muscle. Notably, mitochondrial proteomic analyses show that NUCG or endonuclease G is elevated in mERα^OE^, the protein responsible for generating primers required for DNA polymerase gamma to initiate replication of mtDNA (Figure 5F). Prohibitins (PHBs) 1/2, known ERα targets controlling cristae morphogenesis and mtDNA nucleoid stability,^36^ were also increased in mitochondria from mERα^OE^ vs. control f/f (Figure 5F).

These increases in mtDNA, as well as differences in mitochondrial gene and protein expression in mERα^OE^, were accompanied by a visible change in muscle color to a deep red, compared with control f/f (Figure 5G). Metabolic caging studies show increased whole-body oxygen consumption in male mice harboring a muscle-selective overexpression of *ESR1* (Figures 5H and 5I) Remarkably, male mERα^OE^ mice were refractory to the deleterious effects of HFD, given that fat weight gain was blunted and fasting insulin levels were lower compared with control over the 8 weeks of diet consumption (Figures S7B–S7D). The differences in body composition between the genotypes were not explained by food consumption, ambulatory movement, or alteration of muscle fiber type or structural integrity (Figures S7E–S7H). Moreover, the well-described HFD-induced decrements in glucose tolerance (Figure 5K) and insulin sensitivity (Figures 5L and 5M) observed in control mice were prevented in mERα^OE^ (studies performed in weight matched animals). Collectively, findings point to enhancement of mitochondrial form and function as a key driver of metabolic improvement in skeletal muscle of mice following conditional muscle-selective *ESR1* overexpression.

**Figure 6:**
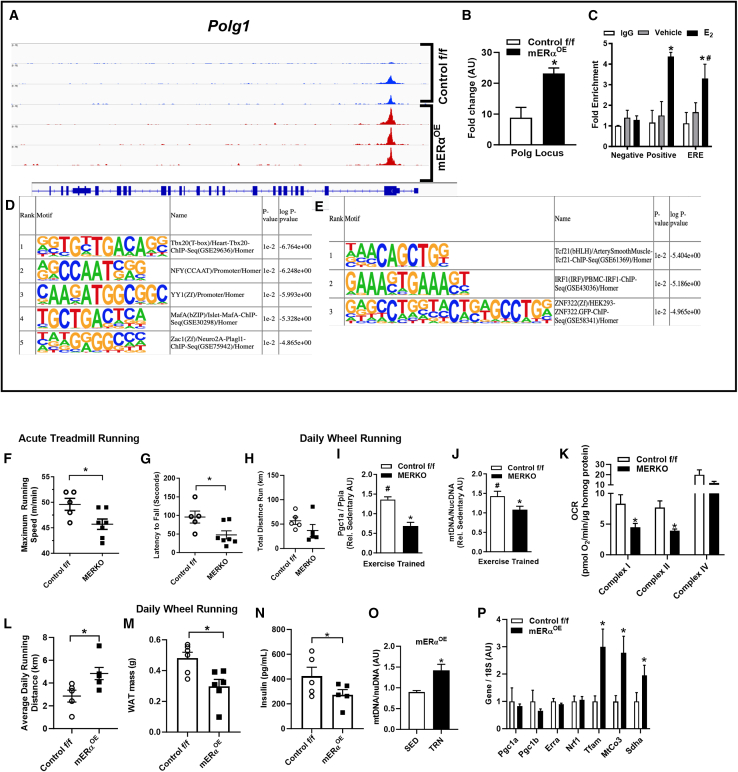
Muscle ERα overexpression reorganizes chromatin structure and enhances exercise-induced adaptation of mitochondria (A and B) Polg1 locus showing peak locations relative to the transition start site (TSS) determined by ATAC sequencing. (C) E_2_-stimulated binding of ERα to the Polg1 proximal promoter (*Pgr*; positive control) (*n* = 3 in triplicate). (D and E) Motif enrichment for top 200 and bottom 200 regions of open chromatin as defined by ATAC sequencing in skeletal muscle of mERα^OE^ mice compared to control f/f. (F and G) (F) Acute exercise by treadmill running to maximum speed and (G) muscular endurance assessed by duration of dynamic hanging (latency to fall test), MERKO vs. control f/f (*n* = 5–7 mice/genotype). (H–J) (H) Total running volume over 30 days of volitional activity was similar between the genotypes, MERKO vs. control f/f (*n* = 6 mice/genotype). However, the well-described exercise training-induced increase in (I) Pgc1a transcript and (J) mitochondrial CN observed for control f/f (open bars, relative to sedentary) was blunted in MERKO (black bars) and not significantly different from SED (*n* = 5/genotype). (K) Complex I and II respiration rates were reduced in frozen muscle homogenates (quadriceps) from MERKO vs. control f/f following training as assessed by Seahorse analysis (*n* = 6/genotype). (L) Average daily volitional running in control f/f (open bars) vs. mERα^OE^ (closed bars) (*n* = 5–6/genotype). (M and N) Adipose tissue mass and fasting insulin concentrations were reduced in mERα^OE^ compared with control f/f mice following the 30-day running intervention (*n* = 6/genotype). (O and P) (O) Mitochondrial DNA CN and (P) transcript expression in skeletal muscle of control f/f (open bars) vs. mERα^OE^ (closed bars) following 30 days of volitional wheel running (*n* = 6/genotype; SED, sedentary; TRN, exercise trained). All values are expressed as means ± SEM detected by Student’s t test. ∗*p* < 0.05 between genotype difference, # within genotype, between conditions comparison.

To determine the impact of *ESR1* expression on global gene accessibility and identify the genomic binding sites that underlie the protection against diet-induced disruption of metabolic health, we performed assay for transposase-accessible chromatin (ATAC) sequencing in nuclei from mice with a muscle-selective overexpression of *ESR1* compared with control. We show that modest *ESR1* overexpression promotes an open chromatin structure that would be necessary for enhanced transcription. We observed an increase in peak area over the *Polg1* transition start site in muscle from mERα^OE^ compared with control f/f (Figures 6A and 6B). Because there is strong evidence linking ERα with *Polg1* expression including the presence of three putative full-consensus estrogen response elements (EREs) in the Polg1 promoter, we performed chromatin immunoprecipitation assays. Compared with the negative and vehicle controls, 17 β-estradiol induced a marked binding of ERα to the proximal promoter of Polg1 (Figure 6C). Estradiol-induced binding of ERα to the proximal promoter of *Polg1* was identical to that of a known ERα-target *Pgr* (positive control; Figure 6C).^37^ On a genome-wide scale, *ESR1* overexpression enhanced an open chromatin structure, and open regions were enriched for Tbx20, NFY, YY1, MafA, and Zac1-binding motifs (Figures 6D and 6E). Specifically, YY1 is associated with regulation of mitochondrial biogenesis, as well as mitochondrial form and function.^38^^,^^39^

Because ERα impacts a wide variety of regulatory gene programs and because *Esr1* overexpression promotes a metabolic phenotype recapitulating many of the molecular adaptations observed in muscle following exercise training (TRN), we investigated the impact of *Esr1* loss and gain of expression in muscle on exercise performance (acute exercise by treadmill running) and metabolic adaptation to daily volitional running (30 days, in cage running wheel). Muscle-specific *Esr1* deletion in MERKO mice reduced running performance (Figure 6F) as well as muscle endurance capacity in response to dynamic hanging (Figure 6G, latency to fall). With respect to volitional exercise training, although MERKO mice performed a similar exercise volume as control mice over the 30-day intervention, the well-described exercise training-induced increase in *Pgc1a* expression and mtDNA CN was blunted in MERKO muscle compared with control f/f (Figures 6H–6J). These findings are consistent with a blunted respiration observed for complex I and II in muscle homogenates from TRN MERKO vs. control f/f mice (Figure 6K). In contrast to MERKO, overexpression of *ESR1* in muscle led to ∼40% increase in daily running distance for mERα^OE^ vs. control f/f (Figure 6L). Surrogate markers of metabolic health, adiposity and fasting insulin levels, were significantly reduced in mERα^OE^ vs. control f/f following the 30-day running intervention (Figures 6M and 6N). Although mtDNA CN was already elevated over control in the basal sedentary (SED) state for mERα^OE^, exercise training stimulated a further increase in mtDNA CN in muscle of mERα^OE^ vs. control f/f (Figure 6O). The exercise-stimulated increase in the mitochondrial genome for mERα^OE^ over control f/f occurred independently of *Pgc1a*, *Pgc1b*, *Erra*, and *NFE2L2/Nrf1*, but was consistent with an increase in mitochondrial transcription factor A, *Tfam1*, as well as expression of mitochondrial (MtCo3) and nuclear transcripts including Sdha, which encodes a subunit complex II that links the electron transport chain and tricarboxylic acid cycle (Figure 6P).

Collectively, our findings confirm that ERα is a critical regulator of muscle oxidative metabolism and insulin sensitivity in both sexes. Specifically, ERα governs elemental aspects of mitochondrial biology including mtDNA replication and mitochondrial nucleoid and cristae structure by modulating fission-fusion dynamics, as well as mitochondrial health surveillance and turnover. *Esr1*-induced improvements in mitochondrial function enhance metabolic health and heighten mitochondrial adaptations to exercise training, thus providing protection against the development of metabolic-related disease.

## Discussion

Herein, we provide evidence that *ESR1* variants in humans are associated with insulin sensitivity assessed by glucose clamp and that ERα action is essential for metabolic health in men and male mice. In human muscle, we determined that there are a high number of transcripts associated with *ESR1* expression, and pathway analysis shows that many of these transcripts encode proteins controlling mitochondrial electron transport chain, mitochondrial respirasome, ATP synthesis-coupled electron transport, as well as muscle architecture and function pathways (sarcomere, myofibril, and contractile fiber, and muscle contractions). Beyond human and murine *ESR1/Esr1*-gene correlations, we determined experimentally that *Esr1/ESR1* exerts direct control over fundamental processes governing mitochondrial form and function, including mitochondrial fission-linked oxidative metabolism of fatty acids in skeletal muscle.^3^ Interestingly, recent work by the NIH MoTrPAC showed that ESR-mediated signaling was one of only 22 pathways enriched across all 6 tissues assessed following 8 weeks of exercise training.^40^ Moreover, exercise-induced ESR signaling was conserved between male and female animals.^40^

Although it is well established that whole-body disruption of estrogen action underlies metabolic abnormalities in humans and mice of both sexes,^2^^,^^3^ the tissues conferring the obesity and insulin resistance phenotypes, as well as the genetic architecture underlying these shifts in metabolism, are incompletely understood. Herein, we provide clinical evidence that *ESR1* expression, in muscle specifically, is predictive of cardiometabolic disease risk in men and women.^21^ We experimentally interrogated the impact of skeletal muscle *Esr1* expression using a Lox-Cre approach with conventional and conditional promoters to generate animals with muscle-selective loss or gain of ERα expression during development and in adulthood.

We employed two different Cre drivers to better understand the impact of *Esr1* on muscle metabolism and insulin action during different life phases. In the current investigation, we provide strong evidence that *Esr1* deletion, whether performed short term in adulthood or in youth, impairs insulin sensitivity. Our findings are independent of sex and are consistent across methods of experimental assessment including *in vivo* glucose clamps, *ex vivo* muscle insulin-stimulated 2-deoxyglucose disposal, and insulin signaling in primary myotubes cultured from KO vs. wild-type mice. The comparative studies between the two *Esr1* KD strategies were designed to resolve inconsistent findings published by Inigo et al. in which short-term deletion (10 weeks) of *Esr1* from muscle of adult female mice failed to induce a detectable impairment in insulin sensitivity (supraphysiological insulin doses, >10–20× that are used herein, were employed by Inigo et al.). Importantly, Inigo et al. did detect a marked elevation in diet-induced hyperinsulinemia and glucose intolerance as well as inflammation in muscle of inducible muscle-specific *Esr1* KO vs. control,^41^ findings typically concordant with skeletal muscle insulin resistance. Collectively, these studies clearly indicate that indeed, *Esr1* expression is required for the maintenance of muscle insulin sensitivity and glucose homeostasis in female and male rodents.

In addition to insulin action, findings from humans and mice show a strong relationship between *Esr1/ESR1* expression and mitochondrial-related pathways. We used comparative transcriptomics in muscle from the HMDP to curate a list of *Esr1*-correlated transcripts that possess ERE-containing promoters. Next, we identified targets altered in expression by E_2_ and differentially expressed in MERKO and mERα^OE^ vs. control f/f. *Polg1* and *Dnm1L* emerged as top candidates. We confirmed direct binding of ERα to the *Polg1* proximal promoter by chromatin immunoprecipitation. Next, we performed KD studies of *Polg1* and *Dnm1L* in C2C12 myotubes, and our data are consistent with a regulatory process by which ERα-Polg1 governs a feedback control of nucleotide metabolism, fission-fusion-mitophagy dynamics, cristae structure, and electron transport chain expression and function. Importantly, the increase in oxidative capacity and insulin sensitivity observed in muscle from *Esr1* overexpression mice was paralleled by increased mtDNA copy and protein expression of a variety of mitochondrial remodeling proteins as well as key signaling factors critical for mtDNA replication and transcription including the fission regulator *Dnm1L*, *Phb* 1/2, and *EndoG*.

Because PHBs stabilize nucleoids where the genome is housed and EndoG directly binds mtDNA driving primer production for replication, our findings point to a role for *Esr1* in the regulation of mtDNA replication and transcription. Indeed, EndoG expression is robustly induced early in training adaptation of muscle (gastrocnemius) from male and female rats, and protein levels remain markedly elevated over 8-week training intervention.^40^^,^^42^ A similar yet delayed proteomic response to exercise training has been observed for Phb1,^40^^,^^42^ and these predominantly mitochondrial-localized proteins are linked with cellular aging and maintenance of muscle mass.^43^^,^^44^ Moreover, expression of inner mitochondrial membrane cristae junction protein Mic60 and distribution of the mitochondrial nucleoids, where mtDNA replication and transcription occurs, govern mitochondrial cristae architecture.^4^^,^^45^^,^^46^ Nucleoid size and clustering, as well as cristae junction width, are associated with metabolic flux and cellular health, although mechanistic links remain inadequately understood. Defects in fission-fusion dynamics disrupt architectural features related to mitochondrial protein compartmentalization and membrane structure, and these mitochondrial phenotypes associate with disease pathobiology.^47^ Findings in *Esr1* and *Drp1* loss and gain of expression models are consistent with their roles in the regulation of oxidative metabolism and mitochondrial fission,^22^ as well as cristae junction composition, nucleoid size,^34^ and mitochondrial genome health.^34^^,^^45^^,^^48^

In addition to reduced *Polg1* in muscle of MERKO, we also observed a diminished expression of the mitochondrial RNA polymerase *Polrmt*, a component of the mitochondrial transcriptional machinery. The binding of *Polrmt* to mitochondrial transcription factor B2 and TFAM is requisite for interaction of the transcriptional heterotrimer with mitochondrial promoter elements. Transcription from light-strand promoters is necessary for mitochondrial gene expression as well as the production of RNA primers required for the EndoG-stabilized initiation of mtDNA replication.^49^^,^^50^ The D-loop region of the mitochondrial genome is anchored to the inner mitochondrial membrane serving as a central functional hub for nucleoid activity; thus, disruption of this regulatory nexus by physical reorganization of cristae is likely to disturb mtDNA replication and transcription. Mitochondrial stress is shown to stall mtDNA replication and promote an enlarged, clustered nucleoid phenotype, similar to that observed in muscle cells devoid of ERα.^51^^,^^52^^,^^53^ Moreover, mitochondrial proteomic analysis of MERKO muscle showed a signature reflective of organelle stress and attempted compensation by mitochondrial ribosomes. Collectively, our findings in both loss- and gain-of-expression mouse models point to a critical role of *Esr1* in the direct regulation of mtDNA replication and transcription with secondary actions impacting the form and function of all major mitochondrial structures.

Considering that increased mitochondrial genome CN and organelle mass in muscle is a hallmark response to daily physical activity,^54^^,^^55^^,^^56^^,^^57^ but that this effect is blunted by impairment of estrogen action,^58^ we determined the impact of muscle *Esr1* expression on adaptations of the mitochondrion to exercise training. Although no difference in mtDNA CN was observed between MERKO and control f/f at baseline in the sedentary state, the signature increase in the genome CN with exercise training observed in control mice was blunted in muscle lacking ERα. That is, mtDNA CN was identical between the MERKO SED and MERKO TRN and not different from SED control. Similarly, a known exercise-responsive transcription factor Pgc1a showed a blunted induction in MERKO TRN vs. SED compared with TRN vs. SED control f/f. In contrast, conditional overexpression of *ESR1* in muscle augmented the training-induced increase in the mtDNA CN, as well as expression of mitochondrial-related transcripts TFAM, MtCO3, and Sdha (response independent of Pgc1α). These observations are consistent with ERα-induced alterations in chromosomal architecture including enrichment of active YY1 sites critical for mitochondrial biogenesis.^38^^,^^39^ These molecular adaptations likely underlie, in part, improvements in exercise performance and training volume seen in male mERα^OE^ mice. The connection between muscle oxidative capacity and central drive for volitional activity remains inadequately understood.

In conclusion, our research identifies *Polg1* as a *bona fide* ERα target gene, and this regulatory nexus governs mitochondrial form and function including architectural remodeling of cristae within the inner mitochondrial membrane. Collectively, our findings suggest that muscle *Esr1* expression is critical for the maintenance of metabolic health and skeletal muscle insulin action in females as well as males and support the notion that ERα can be selectively targeted to combat metabolic dysfunction.

### Limitations of the study

Our research identifies *Esr1/ESR1* as an important determinant of metabolic health and insulin sensitivity in mouse and human. Additional studies in human subjects and genetically engineered rodents are required to understand the range of ERα actions requisite for preservation of mitochondrial form and function that underpin metabolic health. Since the current study relied exclusively on loss and gain of *Esr1* expression mouse models, future work should focus on the specific ERα post-translational modifications and cytosolic binding partners that confer metabolic traits by non-genomic regulation. Although, considering that ERα is a potent nuclear transcription factor governing numerous molecular pathways via direct DNA binding and or transcription factor protein tethering, identification of novel ERα target genes and transcriptional-translational-posttranslational mechanisms of ERα action at rest and during physical activity (e.g., including nuclear receptor crosstalk and unique cofactor complexing) also remain opportune areas of scientific investigation. Given the rise in metabolic disease incidence in the US, novel pharmacological strategies for maintaining ERα action during aging, especially in selective metabolic tissues across short-term life phase transitions, e.g., menopause/andropause, could be leveraged to enhance the healthspan of both women and men.

## Resource availability

### Lead contact

Further information and request for resources and reagents should be directed to and will be fulfilled by the lead contact, Andrea L. Hevener, PhD (ahevener@mednet.ucla.edu).

### Materials availability

All reagents and mouse strains generated in this study are available from the lead contact with a completed materials and animal transfer agreement.

### Data and code availability

• ATAC-seq data have been deposited at GEO and Metabolomics data at MetaboLights and are publicly available as of the date of publication. Accession numbers are listed in the key resources table. All other data reported in this paper will be shared by the lead contact upon request.
• This paper does not report original code.
• Any additional information required to reanalyze the data reported in this paper is available from the lead contact upon request.

## Acknowledgments

We are grateful for the continued support of our research from the Iris Cantor-UCLA Women’s Health Research Center and the UCSD-UCLA Diabetes Research Center. We are thankful for the technical support image analysis by the UCSD Electron Microscopy Core and the sequencing analyses performed by the UCLA UNGC. We are also grateful for the translational research core of the Specialized Center of Research Excellence in Sex Differences and Women’s Health directed by Hooman Allayee (U54DK120342). This work was supported in part by funding from the UCLA Department of Medicine, Iris Cantor-UCLA Women’s Health Research Center and 10.13039/100016206UCLA CTSI (ULTR000124), UCLA Claude D. Pepper Older Americans Independence Center, and the 10.13039/100000002National Institutes of Health (DK060484, P30DK063491, U54DK120342, and NURSA NDSP U24DK097748 to A.L.H.). M.O.G. was also supported by the Eris M. Field Chair in Diabetes Research. M.O.G. and J.I.R. were supported by the 10.13039/100000097National Center for Research Resources, grant/award number: M01-RR00425; 10.13039/100006108National Center for Advancing Translational Sciences, grant/award number: UL1TR000124; 10.13039/100000050National Heart, Lung, and Blood Institute, grant/award number: R01-HL088457; and 10.13039/100000062National Institute of Diabetes and Digestive and Kidney Diseases, grant/award number R01-DK079888. A.J.L. was supported by NIH grants HL28481 and HL30568. UCSD-CMM-EM Core is supported in part by the 10.13039/100000002National Institutes of Health award number S10OD02352.

## Author contributions

Project conceptualization and methodology, A.L.H., V.R., and Z. Zhou; investigation and formal analysis, Z. Zhou, Z. Zhang, V.R., B.D., T.M.M., A.R.S., A.J.L., H.I., N.M., A.M., P.H.T., J.W., T.Q.d.A.V., B.C., M.M., M.Z., M.S., T.S., F.N., B.P., M.P., R.A.-P., K.A.W., S.G., K.R., S.K.M., O.S., and A.L.H. Specifically, J.R.H., B.D., T.J.S., J.A.S., and J.N.A. performed proteomics on mitochondria from muscle of MERKO and mERα^OE^ mice, and S.K.M. performed electron microscopy studies. Project management and data visualization, A.L.H., Z. Zhou, K.R., J.N.A., O.S., A.L.J., M.S., and T.S. M.O.G. and J.I.R. provided human genome-wide association data. Animal resources, K.S.K., S.C.H., and F.J.D. Writing of original draft, Z. Zhou and A.L.H. Writing – review and editing was performed by all authors, and all authors approved the final draft of the manuscript for journal submission.

## Declaration of interests

The authors declare no competing interests.

## STAR★Methods

### Key resources table

**Table:** 

REAGENT or RESOURCE	SOURCE	IDENTIFIER
**Antibodies**		

ERα (MC-20)	Santa Cruz Biotechnology	sc-542; RRID:AB_631470
ERα (IP)	Abcam	ab32063; RRID:AB_732249
ERα	Sigma	06–935; RRID:AB_3097737
GAPDH	Thermo Fisher	MA5-15738; RRID:AB_10977387
*p*-AKTSer473	Cell Signaling Technology	4060; RRID:AB_2315049
AKT	Cell Signaling Technology	4685; RRID:AB_2225340
GLUT4	Sigma	G4048; RRID:AB_1840900
OXPHOS	Abcam	ab110413; RRID:AB_2629281
*p*-Drp1Ser616	Cell Signaling Technology	4494; RRID:AB_11178659
Drp1	Cell Signaling Technology	8570; RRID:AB_10950498
Mff	Abcam	81127; RRID:AB_1860496
Mtfr1	LSBio	LS-C469956-100; RRID:AB_10966406
Fis1	GeneTex	GTX111010; RRID:AB_1950286
Mfn1	NeuroMab	75–162; RRID:AB_NA
Opa1	BD Transduction Laboratories	612606; RRID:AB_399888
Mic60	Abcam	110329; RRID:AB_10859613
Polg	Abcam	128899; RRID:AB_11145308
Pink1	Cayman Chemicals	10006283; RRID:AB_10098326
Parl	Santa Cruz Biotechonology	sc-514836; RRID:AB_2252054
*p*-ParkinSer65	Cell Signaling Technology	sc-36866; RRID:AB_NA
Parkin	Cell Signaling Technology	4211; RRID:AB_2159920
Parkin	Cell Signaling Technology	32833; RRID:AB_3073958
DJ1	R&D Systems	AF3668; RRID:AB_622088
Porin (VDAC)	Cell Signaling Technology	4866; RRID:AB_2272627
LC3B	Cell Signaling	2775; RRID:AB_915950
p62/SQSTM1	Progen	GP62-C; RRID:AB_2687531
Pgc1a	Sigma	3242; RRID:AB_2268462
Phospho Ikka/b (Ser176/180)	Cell Signaling Technology	2697; RRID:AB_2079382
Ikkb	Cell Signaling Technology	2678; RRID:AB_2122301
SAPK/JNK	Cell Signaling Technology	9252; RRID:AB_2250373
Phospho SAPK/JNK (Thr183/Tyr185)	Cell Signaling Technology	4668; RRID:AB_823588

**Bacterial and virus strains**		

ShRNA Esr1	Millipore	TRCN0000026214

**Chemicals, peptides, and recombinant proteins**		

QIAzol Lysis Reagent	Qiagen	79306
Chloroform, HPLC Grade	Thermo Fisher	C606-4
Isopropanol, 99.5%	Acros Organics	32727–0010
Puromycin	Millipore Sigma	P9620
PPT	Tocris	1426
ICI 182, 780 (Fulvestrant)	Tocris	1047
β-estradiol	Millipore Sigma	E−2257
MG132	Millipore Sigma	M8699
Bafilomycin A1	Millipore Sigma	19–148
Cycloheximide	Millipore Sigma	C7698
Insulin	Eli Lily	Humulin R U-100
MitoSOX Mitochondrial Superoxide Indicators	Invitrogen (Molecular Probes)	M36008
Tetramethylrhodamine, Methyl Ester, Perchlorate (TMRM)	Thermo Fisher Scientific	T668
Chloroquine	Millipore Sigma	C6628
Carboxy-H2DCFDA	Invitrogen (Molecular Probes)	C400
Aqueous Glutaraldehyde solution	Electron Microscopy Sciences	16000
Osmium tetroxide	Electron Microscopy Sciences	19100

**Critical commercial assays**		

Cholesterol	In house	N/A
Insulin ELISA	Alpco	80-INSMSU-E01
Glucose	StanBio Laboratory	1071–250
Triglyceride	Sigma Aldrich	TR0100
Phospholipid-C kit	WAKO Diagnostics	997–01801
Glycerol	Sigma Aldrich	FG0100
Qiagen	RNAeasy Kit	74106
Nextera XT DNA Library Preparation Kit	Illumina	FC-131-1024

**Deposited data**		

HMDP transcriptomics	NCBI GEO	GSE64770 PMCID:4349439
MERKO transcriptomics	NCBI BioProject	PRJNA785746
Mitochondrial proteomics	MassIVE	MSV000096257
mERαOE ATACsequencing	This paper	GSE280715
Metabolomics	This paper MetaboLights	Table S1 MTBLS914

**Experimental models: Cell lines**		

C2C12	ATCC	CRL-1772

**Experimental models: Organisms/strains**		

100 strain mouse panel	The Jackson Laboratory	Strain IDs in Table S2A
Esr1 exon 3 lox mouse	Kenneth Korach	JAX #032173
Rosa26-LSL-hERα mouse	Kenneth Korach and Francesco DeMayo	PMCID:6529333 PMCID:4563687
Muscle MCK Cre mouse	The Jackson Laboratory	JAX #006405
Human skeletal actin (HSA)-MerCreMer mouse	The Jackson Laboratory	JAX #025750

**Oligonucleotides**		

Primer sequences	Integrated DNA Technologies (IDT)	Table S3

**Software and algorithms**		

Prism v9	GraphPad Software	https://www.graphpad.com
R Studio Posit Rv4.0.0	R Studio Desktop	https://posit.com
WCGNA		https://cran.r-project.org
DESeq2		https://bioconductor.org/packages/release.bioc/html/DESeq2.html
GTEx data mining via GDCAT	gdcat.org	https://github.com/mingqizh/GD-CAT
GUARDIAN	NIH GWAS Catalog	https://www.ebi.ac.uk/gwas/publications/25524916
Gene Enrichment Analysis		http://pantherdb.org
Graphics	Adobe Illustrator v24.3	http://www.adobe.com
Schematic for graphical abstract	BioRender	www.biorender.com

### Experimental model and study participant details

#### Genome wide association studies

Three cohorts of human subjects (age 32–37 years of age) from MACAD (*n* = 772, 43% male), HTN-IR (*n* = 708, 41% male) and NIDDM-Athero (*n* = 188, 43% male) underwent genome wide association analysis as part of the GUARDIAN Consortium.^59^ Forty-six SNPs near the *ESR1* gene were interrogated for a relationship to insulin sensitivity (Glucose disposal rate; GDR, as assessed by the hyperinsulinemic-euglycemic clamp technique), as previously described.^59^ Two independent signals emerged that were correlated with GDR. The location of these SNPs can be found in Figures S1A and S1B.

#### Genome wide association studies

Three cohorts of Mexican-American subjects (mean age 32–37 years) from MACAD^60^ (*n* = 752, 43% male), HTN-IR^61^ (*n* = 694, 41% male) and NIDDM-Athero (*n* = 182, 42% male) underwent genome wide association analysis as part of the GUARDIAN Consortium.^20^ A total of 1396 SNPs in the *ESR1* gene were interrogated for a relationship to insulin sensitivity using an additive genetic model with adjustment for age and sex. Insulin sensitivity (Glucose disposal rate; GDR) was assessed by the hyperinsulinemic-euglycemic clamp technique, as previously described.^20^^,^^62^ Two independent signals emerged that were correlated with GDR. The location of these SNPs can be found in Figures S1A and S1B.

#### GTEx subjects and analyses using GD-CAT web tool

The Genotype-Tissue Expression (GTEx) is the most comprehensive multi-tissue dataset in humans containing 310 individuals, consisting of 210 male and 100 female (self-reported) participants between the ages of 20–79 years. Gene correlation structure showed strong overlap with known physiologic roles of given endocrine proteins. We have previously shown using the hybrid mouse diversity panel that adopting a gene-centric approach to surveying genetic correlation structure can inform mechanism of coordination between metabolic tissues. All analyses, datasets, and scripts used to generate the associated web tool (GD-CAT) can be accessed via https://github.com/mingqizh/GD-CAT or within the associated docker image. In addition, access to the GD-CAT web tool is also available through the web portal gdcat.org. The portal provides a user-friendly interface for accessing and manipulating the GD-CAT tool without the need for download or installation of any software or packages. The interface and server of the web were built and linked by the shiny package using R (v. 4.2.0).

#### Animals

##### UCLA hybrid mouse diversity panel (HMDP)

All mice were obtained from The Jackson Laboratory and bred at University of California, Los Angeles. Male and female mice were maintained on a chow diet (Ralston Purina Company) until 8 weeks of age when they either continued on the chow diet or were provided a high fat/high sucrose diet (HF/HS Research Diets D12266B; 8 weeks) with the following composition: 16.8% kcal protein, 51.4% kcal carbohydrate, 31.8% kcal fat. A complete list of the strains included in our study is included in Table S2. This resource was established to provide a platform for high resolution genome wide mapping and systems level analysis of gene-gene and gene-trait relationships.

Animal studies were approved by the University of California, Los Angeles Institutional Animal Care and Use Committee. All animal care, maintenance, surgeries, and euthanasia were conducted in accordance with this Institutional Animal Care and Use Committee and the National Institute of Health.

#### Genetically engineered mice

The strategy for the generation of the floxed- Exon 3 ERα (Esr1^f/f^) was previously described.^63^ Floxed mice were crossed with a muscle-specific Cre transgenic mouse (MCK-Cre; The Jackson Laboratory JAX#6475) line or the human alpha actin HSA-MER-CRE-MER tamoxifen inducible line (The Jackson Laboratory JAX#25750^64^ and bred to obtain the following genotypes: MCK-Cre-Esr1^f/f^ (MERKO), Control Esr1^f/f^ CRE negative, and conditional muscle-specific Esr1 knockout (miERα^KO^). ROSA Flox-STOP-ESR1 mice were crossed with a tamoxifen-inducible human α-skeletal actin promoter Cre line to generate animals with a muscle-specific overexpression of human *ESR1* (Table S3; primers for animal genotyping). The generation of mice containing a Cre-dependent inducible human ERα transgene on the Rosa26 locus (Rosa26-LSL-hERα) has also been previously reported.^65^ Briefly, the Myc-FLAG tandem tagged full length human *ESR1* cDNA was cloned into a minigene, containing a CAGGS promoter and a floxed Stop cassette.^66^ The *ESR1*-carrying minigene was inserted into the Rosa26 locus of mouse AB2.2 embryonic stem cells by gene targeting. The targeted AB2.2 cells were initially maintained in the SVJ129-C57BL/6J hybrid and subsequently bred up to a pure BL/6J background. Male and female mice were studied under normal chow and high fat diet (HFD; 8 weeks Research Diets #D12451; 45 kcal% fat) fed conditions. Age and animal sex as indicated in the figure legends and tables.

Control f/f and the muscle-specific *Esr1* engineered mouse lines were maintained on a normal chow (NC) standard diet, and divided into baseline or experimental intervention groups: insulin-stimulated, exercise-trained (30 days of volitional wheel running), high fat-diet fed. Within these categories, mice were further divided into groups by experimental condition, e.g., duration of fasting (6-24h), or *in vivo* (glucose clamps) vs. *ex vivo* (soleus muscle 2-deoxyglucose uptake assays) assessment. All procedures were performed in accordance with the Guide for Care and Use of Laboratory Animals of the National Institutes of Health and were approved by the Animal Subjects Committee of the University of California, Los Angeles.

### Method details

#### Animal characteristics

Blood and tissues were taken from 8weeks to 20-24weeks of age in 6h-fasted mice. Circulating parameters were analyzed at 6 months of age: glucose (Hemocue), insulin, leptin (MSD Mesoscale), and adiponectin (MSD Mesoscale). Glucose tolerance or insulin tolerance tests (GTTs 1000 mg/kg dextrose; ITTs 0.75 U insulin/kg) were performed on 6h-fasted mice, normal chow or high fat diet-fed as indicated.^67^ Exercise performance (treadmill running) and training (30days of volitional in cage wheel running) as previously described.^68^^,^^69^ To test muscular endurance, animals were placed supine and hung from a wire rack to determine the latency to fall.

#### Hyperinsulinemic-euglycemic clamp studies

Two weeks after the GTT, dual catheters were surgically placed in the right jugular vein and glucose clamp studies were performed 3 days post-surgery as previously described.^67^^,^^70^ All animals were fasted for 6 h prior to the clamp and studied in the conscious state. Basal glucose turnover was determined following a 90-min constant infusion of [3-^3^H] D-glucose (PerkinElmer; 5.0 μCi/h, 0.12 mL/h). After the basal period, glucose (50% dextrose, Abbott Laboratories) and insulin (12 mU·kg^−1^·min^−1^, Novo Nordisk Pharmaceutical Industries) plus tracer (5.0 μCi/h) infusions were initiated simultaneously, and glucose levels clamped at euglycemia (∼120 mg/dL) using a variable glucose infusion rate (GIR). At steady state the total glucose disposal rate (GDR), measured by tracer dilution technique, is equal to the sum of the rate of endogenous or hepatic glucose production (HGP) and the exogenous (cold) glucose infusion rate (GIR).^67^^,^^70^^,^^71^ The insulin-stimulated component of the total GDR (IS-GDR) is equal to the total GDR minus the basal glucose turnover rate.

#### Ex-vivo skeletal muscle glucose uptake

Whole muscle *ex vivo* glucose uptake was assessed using 2-deoxy glucose, with minor changes to that described previously.^72^ Briefly, soleus muscles were carefully excised from anesthetized animals and immediately incubated for 30mins in complete Krebs-Henseleit buffer with or without 60μU/mL insulin at 35°C. Muscles were then transferred to the same buffer containing 3 mCi/ml ^3^H-2-deoxy-glucose and 0.053 mCi/ml ^14^C-mannitol and incubated for exactly 20mins. Muscles were then removed from incubation medium, dried, and snap frozen. Muscles were homogenized in lysis buffer and counted for radioactivity and used for immunoblotted for insulin signal transduction. Glucose uptake was standardized to the non-specific uptake of mannitol and estimated as mmol of glucose uptake per gram of tissue.

#### Cell culture and treatments

Cell lines, C2C12 and primary myoblasts, were maintained in high glucose DMEM, 10% fetal bovine serum with penicillin/streptomycin. To obtain myotubes, cells were allowed to reach confluence and switched the media to high glucose DMEM 2% horse serum with penicillin/streptomycin for 5–7 days. MG132 (20 μM), Bafilomycin A1 (25 nM) and cycloheximide (20 μM) were used to block lysosomal degradation and protein synthesis respectively. Three days prior to 10nM β-estradiol, 10 nM 4,4′,4′ –(4-Propyl-[1H]-pyrazole-1,3,5-triyl)trisphenol (PPT) and 1μM ICI 182,780 treatments, cells were cultured in phenol red free DMEM with 2% charcoal-treated horse serum with penicillin/streptomycin.

### Lentiviral-induced ERα knockdown in C2C12 myocytes

To achieve *Esr1* knockdown (KD), lentiviral particles (Sigma) carrying shRNA targeted to ERα were used to transduce C2C12 myoblasts. After selecting positive transformants with puromycin (5ug/ml), the selected clones were expanded and analyzed for knockdown (KD) efficiency as measured by qPCR and immunoblotting. The resulting cultures were then used for subsequent assays in undifferentiated and differentiated states as published.^3^

#### Mitochondria isolation from muscle

Mitochondria isolation from muscle tissue was performed following instructions provided in the Mitochondria Isolation Kit for Tissues (Thermo Scientific, 89801). 200 mg of muscles were cut into small pieces and pre-treated with trypsin and pelleted by quick centrifugation. Tissue was disrupted by 20 dounce strokes and centrifuged at 1,000 ∗g for 3 min at 4°C. Supernatants were further centrifuged at 12,000 ∗g for 15 min to acquire the mitochondrial fraction as a pellet and cytosolic fraction as supernatant. All samples were stored at −80°C for subsequent analysis.

#### Seahorse respirometry studies

Respirometry was performed on myocytes as well as frozen muscle as previously described (RIFS).^3^^,^^73^ Briefly, mouse quadriceps muscle was pulverized in liquid nitrogen and subjected to frozen tissue respirometry as described.^73^^,^^74^^,^^75^ Frozen tissues were thawed in ice-cold PBS and homogenized in MAS buffer (70 mM sucrose, 220 mM mannitol, 5 mM KH_2_PO_4_, 5 mM MgCl_2_, 1 mM EGTA, 2 mM HEPES pH 7.4). Muscle was mechanically homogenized with 10–20 strokes in Teflon-glass homogenizer. All homogenates were centrifuged at 1,000 *g* for 10 min at 4°C and the supernatant was collected for protein concentration determination by BCA (Thermo Fisher).

Tissue lysate homogenates (skeletal muscle, 6 μg) were loaded into Seahorse XF96 microplate in 20 μL of MAS. The loaded plate was centrifuged at 2,000 *g* for 5 min at 4°C (no brake) and an additional 130 μL of MAS containing cytochrome *c* (10 μg/mL, final concentration), was added to each well. Substrate injection was as follows: pyruvate + malate (5 mM each), NADH (1 mM), or 5 mM succinate + rotenone (5 mM + 2 μM) were injected at port A; rotenone + antimycin A (2μM + 4 μM) at port B; TMPD + ascorbic acid (0.5 mM + 1 mM) at port C; and azide (50 mM) at port D. These conditions allow for the determination of the respiratory capacity of mitochondria through Complex I, Complex II, and Complex IV.

With respect to the performance characteristics of frozen respirometry (RIFS) vs. studies in fresh tissue or cells, the RIFS assay was validated in a side-by-side comparison of fresh vs. frozen tissue analysis performed in samples collected from humans and mice (brown adipose tissue, liver heart, and skeletal muscle).^3^^,^^73^ The RIFS assay recapitulates the results obtained with fresh tissues given that the frozen respirometry approach preserves 90–95% of the maximal respiratory capacity of the tissue. Findings from the RIFS approach also show the retention of differences in mitochondrial respiration in the context of genetic mutation and treatment with compounds known to stimulate or reduce respiration. Moreover, RIFS recapitulates physiologically relevant differences in OXPHOS capacity across a variety of tissues.^3^^,^^73^

#### Reactive oxygen species analysis

Primary myotubes were washed and incubated in low glucose DMEM at 37°C, 5%CO_2_ in the dark with 25 μM of Carboxy-H_2_DCF-DA (Molecular Probes), washed with PBS and incubated 15 min with 5μM of mitoSOX (Molecular Probes), washed and quickly trypsinized, and pelleted and retained on ice. Cells were resuspended in FACS buffer (PBS 3% BSA) with DAPI (25 μg/mL) and analyzed immediately by flow cytometry on an LSRII (Becton Dickinson) with FlowJo software (Treestar Inc). Unstained and single stained cells were used for establishing compensation and gates, and only live cells (DAPI negative) were analyzed.

#### Electron microscopy

Muscles were harvested and immediately immersed in 2% glutaraldehyde in phosphate-buffered saline for 2h at room temperature and then at 4°C overnight. Fixed tissues were washed and postfixed in a solution of 1% OsO_4_ for 2 h. After further washes in buffer, tissues were dehydrated through serial immersions in graded ethanol solutions (50–100%). passed through propylene oxide and infiltrated in mixtures of Epon 812 and propylene oxide 1:1 and then 2:1 for 2 h each and then in pure Epon 812 overnight. Embedding was then performed in pure Epon 812 and curing was done at 60°C for 48h. Muscle longitudinal sections of 60 nm thickness were cut using an ultramicrotome (RMC MTX). The sections were double-stained in aqueous solutions of 8% uranyl acetate for 25 min at 60°C and lead citrate for 3 min at room temperature. Thin sections were subsequently examined by the UCSD CMM-EM Core as previously described.^3^

#### Mitochondrial proteomics

Mitochondria were lysed and samples were centrifuged for 10 min at 4°C and 18 000 rcf to remove debris. Protein extraction and peptide analysis was performed by the Pacific Northwest National Laboratory (PNNL) as previously described.^76^

#### DNA & RNA extraction, cDNA synthesis, quantitative RT-PCR, microarrays, and RNAsequencing

DNA and RNA were extracted from a homogeneous portion of frozen quadriceps muscle homogenate using DNeasy/RNeasy Isolation kits (Qiagen) as described by the manufacturer. Isolated DNA and RNA was tested for concentration and purity using a NanoDrop Spectrophotometer (Thermo Scientific). Isolated RNA was converted into cDNA, checked for purity, and qPCR of the resulting cDNA levels was performed as previously described.^77^ All genes were normalized to the housekeeping gene Ppia or 18S. Mitochondrial DNA content was assessed as a ratio of mitochondrial DNA (mtCO3) to nuclear DNA (SDHA). See Table S3 for a list of qPCR primers. Total RNA from HMDP mouse muscle (211 females, 228 males) was hybridized to Affymetrix HT_MG-430A arrays and scanned using standard Affymetrix protocols. To reduce the chance of spurious association results, RMA normalization was performed after removing all individual probes with SNPs and all probesets containing 8 or more SNP-containing probes, which resulted in 22,416 remaining probesets.

#### Metabolomics

Muscle samples, n = 5–8 per genotype per sex (8 months of age), were prepared using the automated MicroLab STAR system from Hamilton Company. Several recovery standards were added prior to the first step in the extraction process for QC purposes. To remove protein, dissociate small molecules bound to protein or trapped in the precipitated protein matrix, and to recover chemically diverse metabolites, proteins were precipitated with methanol under vigorous shaking for 2 min (Glen Mills GenoGrinder 2000) followed by centrifugation. The resulting extract was divided into five fractions: two for analysis by two separate reverse phase (RP)/UPLC-MS/MS methods with positive ion mode electrospray ionization (ESI), one for analysis by RP/UPLC-MS/MS with negative ion mode ESI, one for analysis by HILIC/UPLC-MS/MS with negative ion mode ESI, and one sample was reserved for backup. Samples were placed briefly on a TurboVap (Zymark) to remove the organic solvent. Sample extracts were stored overnight under nitrogen before preparation for analysis.

##### Ultrahigh performance liquid chromatography-tandem mass Spectroscopy (UPLC-MS/MS)

A Waters ACQUITY ultra-performance liquid chromatography (UPLC) and a Thermo Scientific Q-Exactive high resolution/accurate mass spectrometer interfaced with a heated electrospray ionization (HESI-II) source and Orbitrap mass analyzer operated at 35,000 mass resolution were used for these analyses. Principal component analysis (PCA) was performed to obtain a high-level view of the dataset. The experimental groups (and their respective controls) showed good separation in the PCA, though only male (Control fl/fl and MERKO) groups displayed further separation. Hierarchical clustering analysis (HCA) was performed to observe the structure of the data by grouping samples together by genotype. Random forest analysis (RFA) aided in the identification of biomarkers differentiating classification groups. RFA assessing the effect of muscle ERα deletion in males yielded a moderate predictive accuracy of 67% (random chance would be expected to yield 50%). RFA highlighted biochemicals involved in redox homeostasis (e.g., *glutathione, methionine sulfoxide, cysteine, cystine*), and phospholipid metabolism (e.g., *2-palmitoleoyl-GPC, 1,2-dilinoleoyl-GPE, 1-palmitoleoyl-GPC*), and carbohydrate metabolism (e.g., *ribose 5-phosphate, mannose, maltotriose*). Table S1. Data deposited at MetaboLights as indicated in Key Resources and in Data Availability sections.

#### Immunoblot analysis

Mouse tissue samples were pulverized in liquid nitrogen and homogenized in RIPA lysis buffer containing freshly added protease (complete EDTA-Free, Roche) and phosphatase inhibitors (Phosphatase Inhibitor Cocktail 2, Sigma). All lysates were clarified, centrifuged, and resolved by SDS-PAGE. Samples were transferred to PVDF membranes and subsequently probed with the following antibodies for protein and phospho-protein detection: ERα (Santa Cruz Biotechnology), Mfn1 (N111/24, Neuro Mab), GLUT4 and Mfn2 (Sigma-Aldrich), GAPDH (Millipore), pan actin, t-Akt/*p*-AKT^Ser473^, IKK-β/p-IKKα/β^Ser180/181^, JNK/p-JNK^Ser183/Tyr185^, LC3B, tDRP1/p-DRP1 ^Ser616/637^ (Cell Signaling), FIS1 (Genetex), OPA1 (BD Biosciences), MitoProfile OXPHOS (MS604/ab110413; CI-NDUFB8 20kDa, CII-SDHB 30kDa, CIII-UQCR2 Core protein 2 48 kDa, CIV-MTCO140 kDa, and CV-ATP5A 55kDa) and Porin (Mitosciences, Abcam), PINK1 (Cayman Chemicals), p62 (Progen Biotechnik GmbH), and Parkin, GFP (Abcam). Densitometric analysis was performed using BioRad Quantity One image software.

#### Chromatin immunoprecipitation

Stable ERα-expressing C2C12 myotubes (described above) were used for ChIP experiments. Cells were grown to confluence and at day 3 of differentiation were treated for 60 min with vehicle or 10 nM β-estradiol. Cells were harvested and ChIP-PCR analyses were performed as previously described^12^^,^^78^ using an antibody against ERα (ab32063, Abcam). Primers for detection of the *Polg1* promoter by qPCR are listed in the Table S3.

#### ATAC sequencing

Nuclei were isolated from skeletal muscle of normal chow-fed, Control f/f and mERα^OE^ mice at 4 months of age (*n* = 3/genotype). Briefly, 50 mg of frozen gastrocnemius muscle was minced and dounced in NIB buffer (20mM Tris-HCl, 50mM EDTA, 5mM Spermidine, 0.15mM Spermine, 0.1% mercaptoethanel, 40% glycerol pH = 7.5) for 120 strokes. Homogenize samples were incubated on ice for 5 min and centrifuged at 11,00 g at 4°C. The supernatants were filtered through 70 μm nylon mesh and washed with RSB buffer (10mM Tris-HCl, 10mM NaCl, 3mM MgCl_2_ pH7.4). ATAC-seq libraries were prepared from 50,000 nuclei using the Nextera Tn5 Transposase and DNA library preparation kit (Illumina) as described with slight modifications.^79^ Libraries were pair-end sequenced (50bp) on NovaSeq SP2. Reads were mapped to the mouse genome (NCBI37/mm9) using Bowtie2.^80^ Duplicated reads were removed, mapped to the mitochondrial genome, or aligned to unmapped contiguous sequences. MACS2 was employed to perform peak calling.^81^ The average RPKM from 3 replicates was used to quantify the accessibility across all called-peaks. Peak visualization was performed by Integrative Genomics Viewer (IGV).^82^

### Quantification and statistical analysis

#### Seahorse data analysis

Wave software (Agilent) was used to export OCR rates normalized by protein and MitoTracker Deep Red to GraphPad Prism v7.02. Complex I-, II-, and IV-dependent respiration was calculated by subtracting OCR values after injection of inhibitors from the substrate-induced maximal OCR.^73^

#### Gene Set Enrichment Analysis

Gene Set Enrichment Analysis (GSEA) is a rank-based approach that determines whether any *a priori* defined gene sets, including genes involved in the same biological process or participating in the same pathway, display concordant behavior. FGSEA (specifically, FGSEA*-multi-level*) is a fast implementation of gene permutation GSEA that is based on an adaptive multi-level split Monte Carlo scheme for the estimation of arbitrarily-small *p*-values. It requires gene-level statistics, called ranking metric values, and a collection of gene sets as input. Signed -log10-transformed *p*-value (calculated from the differential analysis results for each contrast) was selected as the ranking metric, where the sign indicates the direction of the log2 fold-change. Although similar to the t-statistic, this metric better separates extreme values from those closer to zero. For each contrast, ranking metric values were calculated at the level of individual features (i.e., proteins, transcripts, metabolites). Proteomics and transcriptomics values were aggregated to the Entrez gene ID level by taking the arithmetic mean, and any features that did not map to an Entrez gene were discarded prior to analysis.

#### Statistics

Values presented are expressed as means ± SEM. Statistical analyses were performed using Student’s t test when comparing two groups of samples or one-way analysis of variance (ANOVA) with Tukey’s post hoc comparison for identification of significance within and between groups using SPSS (graduate pack, Chicago, IL) or GraphPad Prism 5 (GraphPad Software, San Diego). Mean differences between groups over time were assessed by repeated measures analysis of variance. Significance was set *a priori* at *p* < 0.05.

### Additional resources

#### Data availability

The RNAsequencing data presented in this publication have been deposited in NCBI’s Gene Expression Omnibus and are accessible through GEO Series accession number GSE103722, GSE64770/GSE103722 (https://www.ncbi.nlm.nih.gov/geo/query/acc.cgi?acc=GSE103722) and NCBI BioProject ID PRJNA785746. Mitochondrial proteomics are available at MassIVE MSV000096257, metabolomics in Table S1, and ATACsequencing at NCBI GEO:GSE280715. All GTEx analyses, datasets and scripts used to generate the associated web tool (GD-CAT) can be accessed via: https://github.com/mingqizh/GD-CAT. Access to the GD-CAT web tool is also available through the web portal gdcat.org. This portal was created to provide a user-friendly interface for accessing and using the GD-CAT tool without the need to download or install any software. Metabolic tissue data was accessed through GTEx V8 downloads portal as previously described.^83^ Metabolomics data has been deposited at MetaboLights, study number MTBLS914, and accessed at: https://urldefense.com/v3/__https://www.ebi.ac.uk/metabolights/MTBLS914__;!!F9wkZZsI-LA!EJIAwtDUDpAAUxeonUro-L3xmTwYSGZneknf3hAiQc83MPO6kUs1W16Y2LJVaEV_4Sq46H30dKNI8mNTrP0ZOScWwRB2$%5Bebi%5B.%5Dac%5B.%5Duk%5D.

Data are publicly available as of the date of publication. Accession numbers are also listed in the key resources table.

## Supplemental information

Document S1. Figures S1–S7 and Table S3

Table S1. Metabolomics data for male MERKO and mERαOE mice

Table S2. Hybrid Mouse Diversity Panel (HMDP) mouse strains, experimental animal numbers, and JAX animal acquisition # for each strain of mouse studied as part of the HMDP

Document S2. Article plus supplemental information
