# Supplementary material for: Muscle metabolic resilience and enhanced exercise adaptation by Esr1-induced remodeling of mitochondrial cristae-nucleoid architecture in males
Source: Cell Rep Med. 2025 May 5;6(5):102116. doi: 10.1016/j.xcrm.2025.102116 (PMC12147913; doi:10.1016/j.xcrm.2025.102116)
Supplement: Document S1. Figures S1–S7 and Table S3 [file mmc1.pdf]

Cell Reports Medicine, Volume *6*

Supplemental information

Muscle metabolic resilience and enhanced exercise adaptation by Esr1-induced remodeling of mitochondrial cristae-nucleoid architecture in males

Zhenqi Zhou, Timothy M. Moore, Alexander R. Strumwasser, Vicent Ribas, Hirotaka Iwasaki, Noelle Morrow, Alice Ma, Peter H. Tran, Jonathan Wanagat, Thomas Q. de Aguiar Vallim, Bethan Clifford, Zhengyi Zhang, Tamer Sallam, Brian W. Parks, Karen Reue, Orian Shirihai, Rebeca Acin-Perez, Marco Morselli, Matteo Pellegrini, Sushil K. Mahata, Frode Norheim, Mingqi Zhou, Marcus M. Seldin, Aldons J. Lusis, Cathy C. Lee, Mark O. Goodarzi, Jerome I. Rotter, Joshua R. Hansen, Ben Drucker, Tyler J. Sagendorf, Joshua N. Adkins, James A. Sanford, Francesco J. DeMayo, Sylvia C. Hewitt, Kenneth S. Korach, and Andrea L. Hevener

Locus plots of ESR1 - SNPs associated with insulin sensitivity by glucose clamp

a b

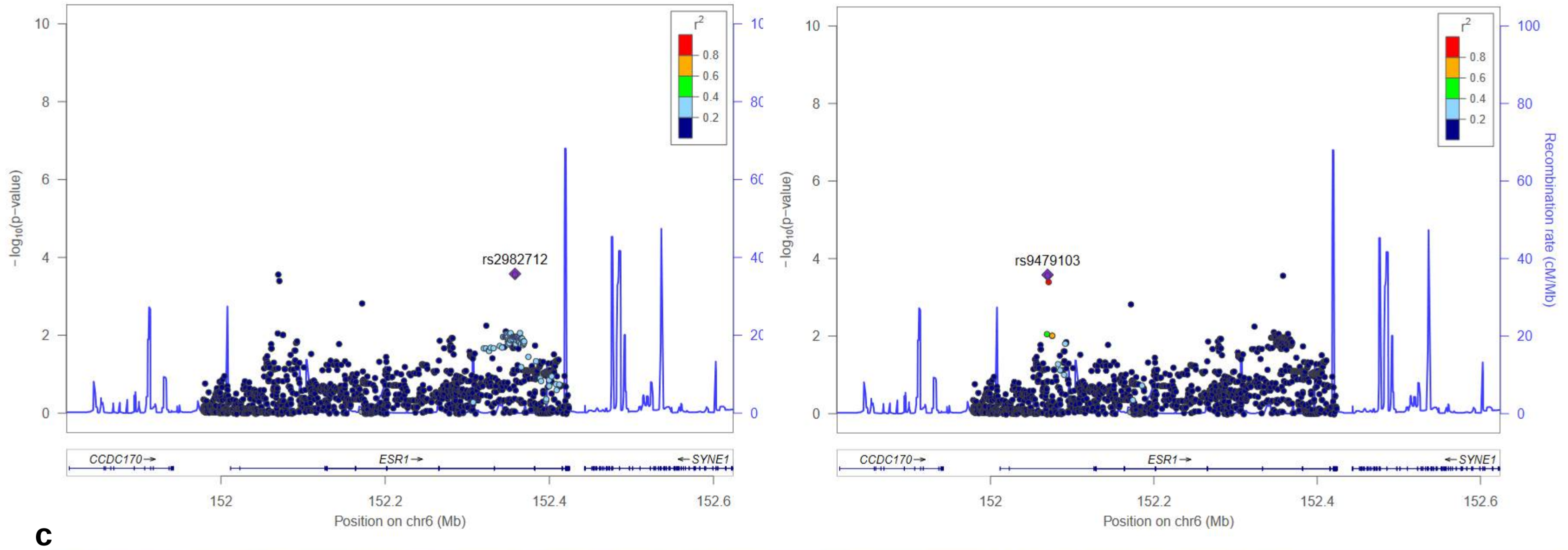

c

ESR1 SNPs	Research Study	Clinical Trait	Transcription Factor Binding
rs2982712	Insulin Resistance Atherosclerosis Family Study	Insulin Sensitivity	Barx2, Dlx5, E2F, Gbx2, Mxs2, Nkx1-1, Zbtb3
rs9479103	Insulin Resistance Atherosclerosis Family Study	Insulin Sensitivity	Foxa
rs9479126	WHI SHARe	Diabetes Risk	CTCF, GR, Rad21, TATA, YY1
rs17081685	WHI SHARe	Diabetes Risk	Foxm1, YY1
rs1856057	WHI SHARe	Diabetes Risk	MEF2, SOX, TATA, TFIIA

Zhou et al. Supplemental fig. S1

a

Top 30 Transcripts Significantly Associated with ESR1	BICOR (P<0.001)
EML1	0.858
KLHL33	0.828
MYOZ3	0.826
DIXDC1	0.812
RASGRP3	0.811
PPP2R3A	0.811
MDFIC	0.808
PPP1R3A	0.807
RASL12	0.806
FILIPL	0.804
NR3C2	0.804
PCYOX1	0.803
PRR33	0.797
RCSD1	0.797
BHLHE41	0.794
RBFOX2	0.793
TMEM182	0.792
CNNM4	0.79
FSD2	0.787
ABCB4	0.785
SEMA6D	0.784
LRRC39	0.783
KBTBD13	0.780
ASB15	0.777
FRAS1	0.777
ADAM19	0.776
APBA1	0.774
SYNE1	0.773
KLHL13	0.773
SBK1	0.773

b

GSEA pathways from ESR1 positive and negative transcript correlations
Skeletal Muscle - Men

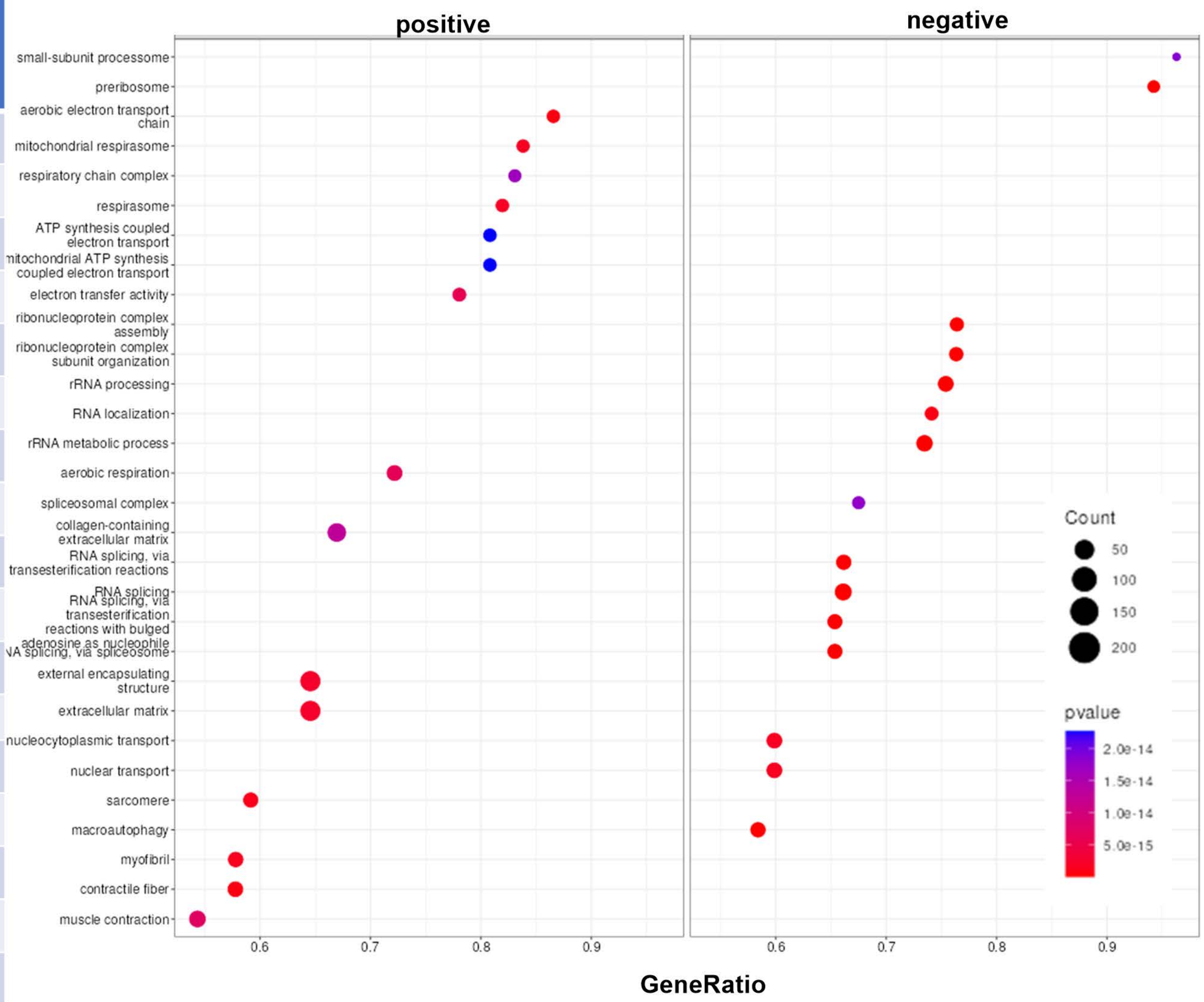

Zhou et al. Supplemental fig. 2

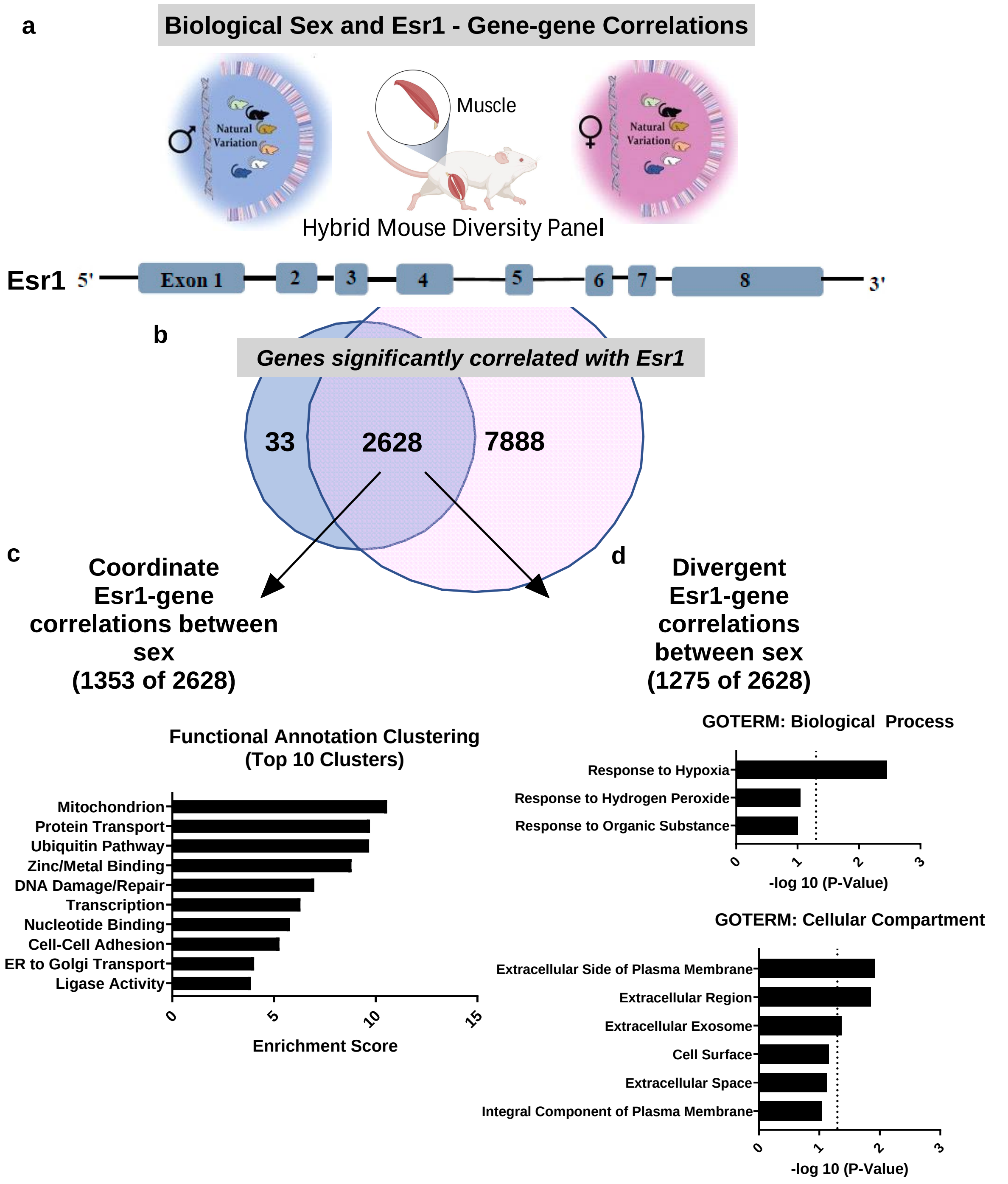

Zhou et al. Supplemental fig. S3

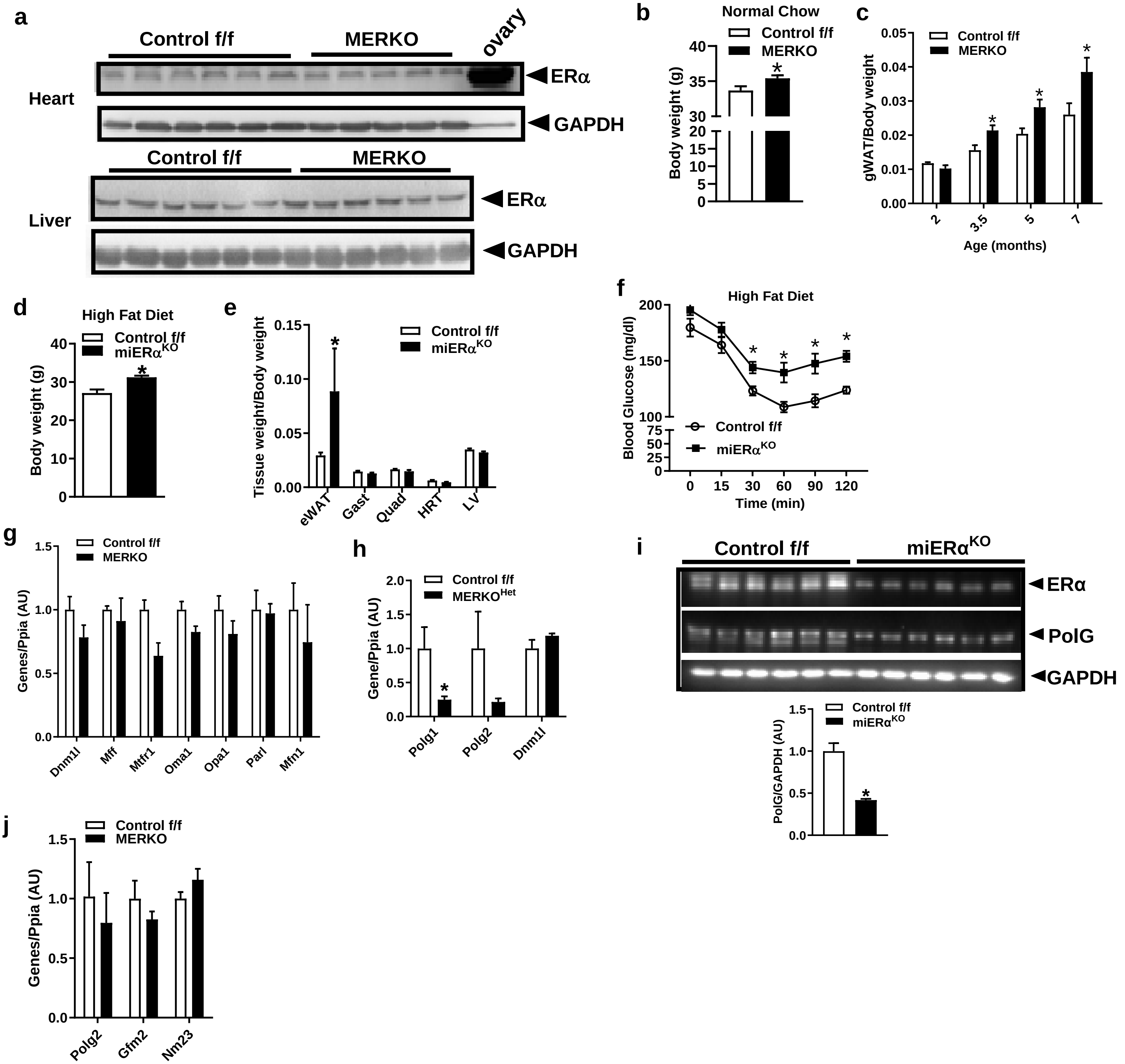

Zhou et al. Supplemental fig. S4

Mitochondrial Proteomics

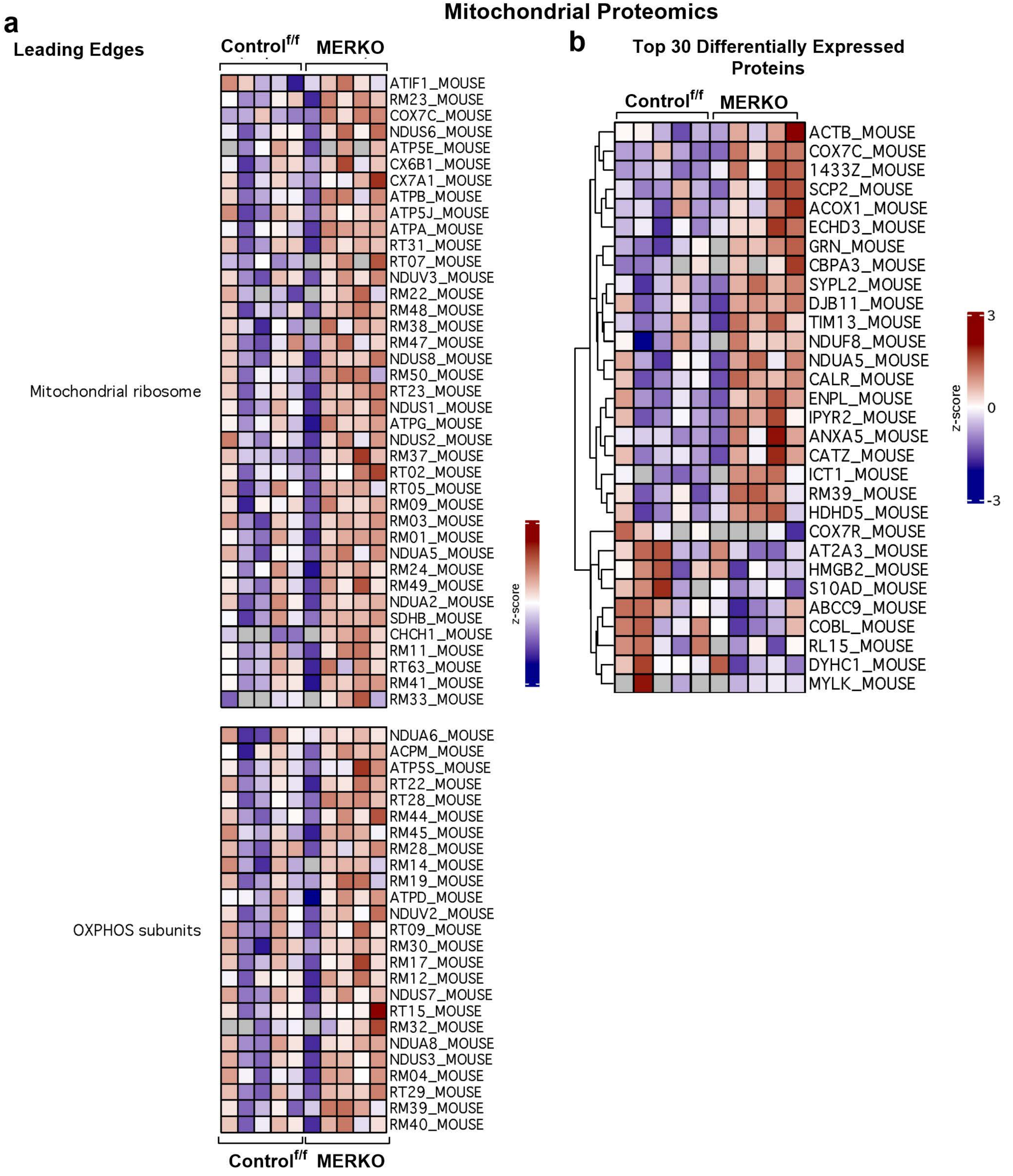

Zhou et al. Supplemental fig. S5

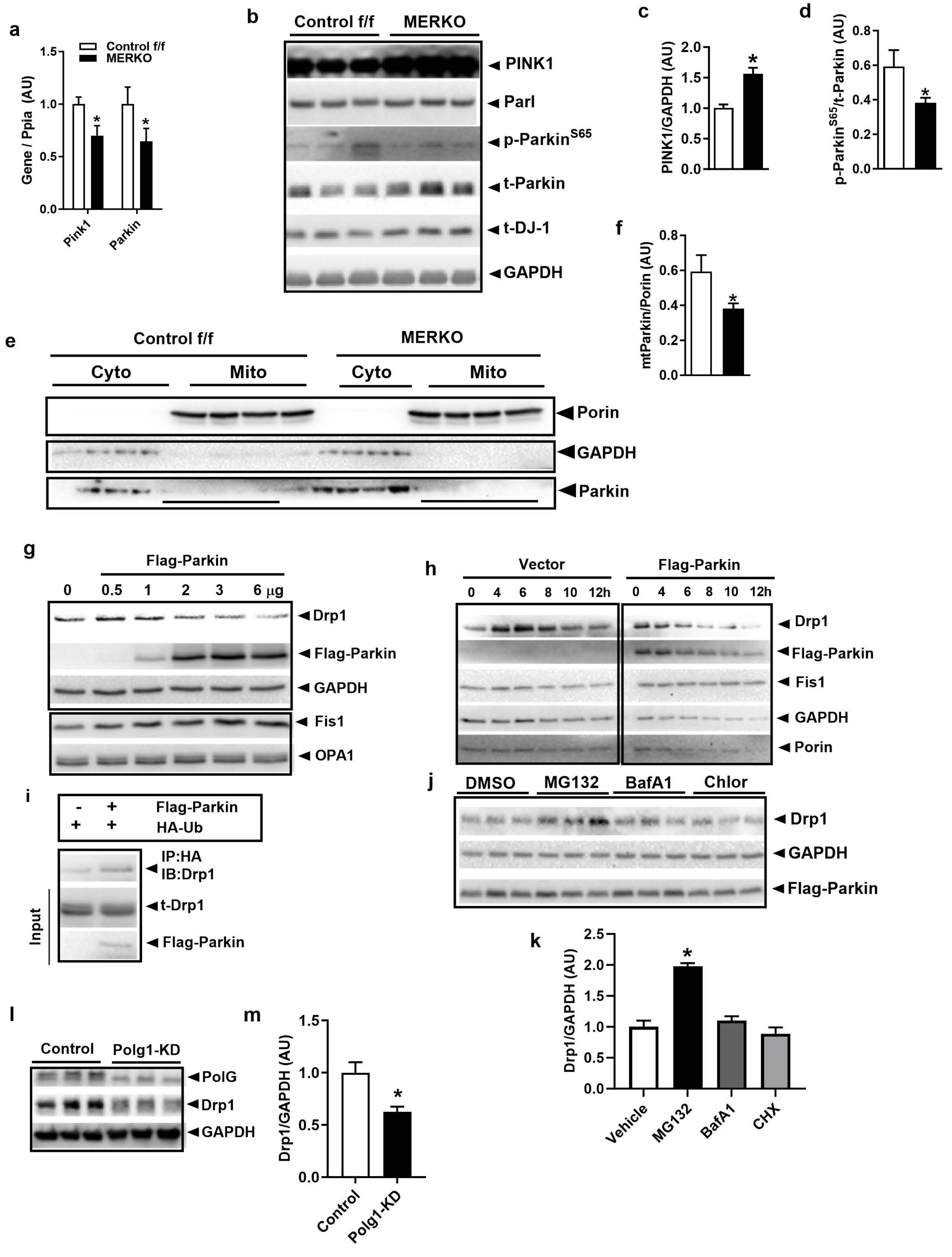

Zhou et al. Supplemental fig. S6

Studies in Male Muscle-specific Conditional ERα Overexpression Mice

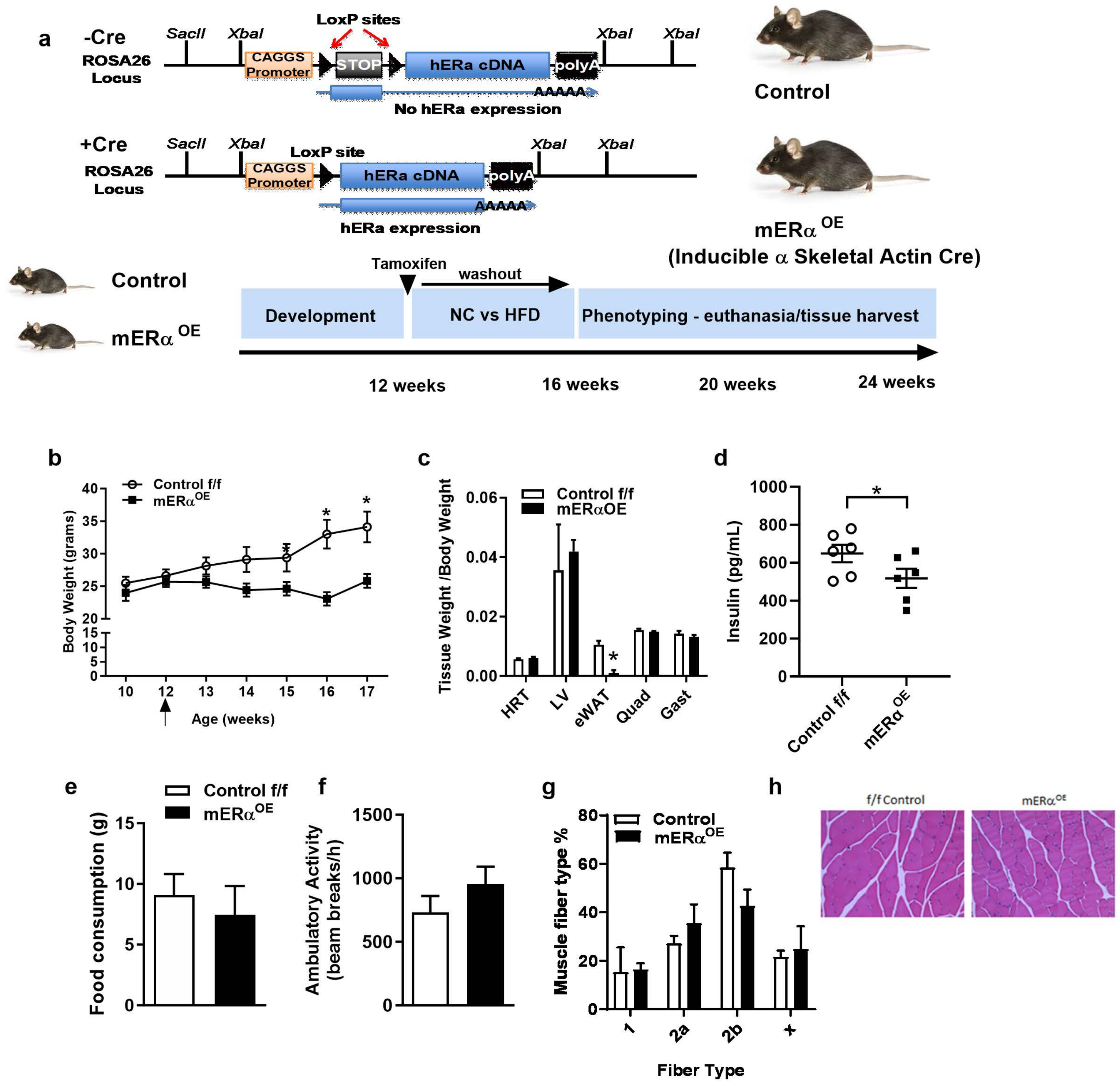

Zhou et al. Supplemental fig. S7

Supplemental table S3. Primer list for qPCR and ChIP analyses.

Gene Name	NCBI Ref.Seq	Forward	Reverse
Acaca	NM_133360	CTGAAGCAGATCCGCAGCTT	GGTGAGATGTGCTGGGTCATG
Acacb	NM_133904	CATACACAGAGCTGGTGTTGGACT	CACCATGCCCACCTCGTTAC
Acadm	NM_007382	GCAGCCAATGATGTGTGCTTAC	CACCCTTCTTCTCTGCTTTGGT
Acox1	NM_015729	GCCCAACTGTGACTTCCAT	GGCATGTAACCCGTAGCACT
Ankrd1	NM_013468.3	GGAACAACGGAAAAGCGAGAA	GAAACCTCGGCACATCCACA
Atp5A1	NM_007505	TCTCCATGCCTCTAACACTCG	CCAGGTCAACAGACGTGTCAG
Cox7a1	NM_009944	CTCTTCCAGGCCGACAATGA	CCCAGCCCAAGCAGTATAAGC
Cpt1b	NM_009948	CTCCTGGAAGAAACGCCTTATT	CACCTTGCAGTAGTTGGAACC
Cyc1	NM_025567	TGGCTCCTCCCATCTACACAG	CGATGGTCATGCTCTGGTTCT
Cycs	NM_007808	CCAAATCTCCACGGTCTGTTC	ATCAGGGTATCCTCTCCCCAG
Dnm1L	NM_001025947	CGTGGACTAGCTGCAGAATG	TGCCTCAGATCGTCGTAGTG
Enox2	NM_001271447.1	TGCAACAGCATCTGCTCAAAG	TGCACACAGTCTAGAACCACA
Err1	NM_007953	GGAGGACGGCAGAAGTACAAA	GCGACACCAGAGCGTTCAC
Errg	NM_011935	TTGTACTTCTGCCGACCTCC	TGAGATCACAAAGCGCAGAC
Esr1 (ex 3)	NM_007956	GCCAGAATGGCCGAGAGAG	CCCCATAATGGTAGCCAGAGG
Esr1(ex4-5)	NM_007956	GCTACTGTGCCGTGTGCAA	TGTCAATGGTGCATTGGTTTG
Esr2	NM_010157	GCCAACCTCCTGATGCTTCT	TCGTACACCGGGACCACAT
Fabp4	NM_024406.2	TTCGATGAAATCACCGCAGA	GGTCGACTTTCCATCCCACTT
Fasn	NM_007988	TGCTCCCAGCTGCAGGC	GCCCGGTAGCTCTGGGTGTA
Fbxo32	NM_026346	TCAGGGATGTGAGCTGTGAC	AAGGAGCGCCATGGATACT
Fis1v2	NM_001163243	AGGAGCTGGAACGCCTGATTGATA	AGGATTTGGACTTGGAGACAGCCA
Fkbp5	NM_010220.4	GATGAGGGCACCAGTAACAATG	CAACATCCCTTTGTAGTGGACAT
Gfm2	NM_177266.5	ACCGTCCAACACCCTCAAG	AAGAACGAGAAAGGGGCATT
Gper	NM_029771 NM_008161	CCAAGCCTCAACACTCACACA GATGGTGAGGGCTCCATACT	CCTGGGAGCCTGTTAGTCTCA CATCCTG[illegible]
Gpx3	NM_144799	ATGGCAAAAGTGGCTAAGGA	TTGCATATTTTCCTCCACGA
Mef2c	NC_005089	GCAGGATTCTTCTGAGCGTTCT	GTCAGCAGCCTCCTAGATCATGT
Mff	NM_029409.3	GCAGTTGGCAGGCTAAAAAG	TCAGGTAGCATATGGGGAGG
Mfn1	NM_024200	GCTTCCGACGGACTTACAAC	TGAATAACCGTTGGGATGCT
Mfn2	NM_133201	ATTGATCACGGTGCTCTTCC	GTCCTGGACGTCAAAGGGTA
mt-Co3	NM_145518	CACTCGTTCCACCTCAGCTA	GACGGCTCCTCTACTGCCT
Ndufs1	NM_010938	GAACTGCCAACCACAGTCAC	CGTCTGGATGGTCATTTCAC
Nrf1	NM_020569	AACACACCCACTGGCTAAGG	GTGCCTCCACAATGGCTAGT
Opa1	NM_133752	TCCTGGTGAAGAGCTTCAATG	TTTGCAGAAGACGGTGAGAA
Park2	NM_016694	ATCGACCTCCACTGGGAAG	GCGTAGGTCCTTCTCGACC
Park6	NM_026880.2	GGATGTCGTCCTGAAGGGAG	GCTTCGCTGGAGGAACCTG
Park7	NM_181414	GTTCAGCTTCATCGGAGGAG	ATTCTTGGAGTTGGAGACCG
Pik3c3	NM_017462	TAGCTGGCTGGTCCAAGAGT	CGACGTGGAGGTCTGCTT
Pim1	NM_008842.4	ACTTTGATGGGACCCGAGTG	CCTGAAGAACACTTGGCCCT
Polg	NM_017462.2	TAGCTGGCTGGTCCAAGAGT	CGACGTGGAGGTCTGCTT

Polg1	**NM_011145**	**GCCTCGGGCTTCCACTAC**	**AGATCCGATCGCACTTCTCA**
Polg2	**NM_015810**	**CCGTTTTCCAGCGTAGTCTC**	**TTCTGTGTGGCCTGGCTATT**
Polrmt	**NM_172551.3**	**CTCATCTCAGGTGTGCCCTC**	**TCTGCAGCTCAAGAAGGAGC**
Ppard	**NM_011146**	**GCCCTTTGGTGACTTTATGG**	**CAGCAGGTTGTCTTGGATGT**
Pparg	**NM_008904**	**TGAGGACCGCTAGCAAGTTT**	**TGAAGTGGTGTAGCGACCAA**
Ppargc1	**NM_133249**	**CTGAGTCAAAGTCACTGGCG**	**GCTCTCGTCCTTCTTCCTCA**
Ppargc1b	**NM_008907**	**AGCCAAATCCTTTCTCTCCAG**	**CACCGTGTTCTTCGACATCA**
Ppia	**NM_023281**	**TACTACAGCCCCAAGTCT**	**TGGACCCATCTTCTATGC**
Ppp1r3c	**NM_016854.2**	**GCTCATTCACCACCTCTGAAG**	**GCTTGACACTGAGACATGGTT**
Sdha	**NM_009204**	**CCCCCGATACCTCTACATCATC**	**GCATCAGACACATCAGCCCAG**
Sirt1	**NM_019812**	**ACACAGAGACGGCTGGAACT**	**AGACCTCCCAGACCCTCAAG**
SLC2a4	**NM_011018**	**TTTCTGGGGTAGTGGGTGTC**	**CTGAAGAATGTGGGGGAGAG**
Sod2	**NM_013671**	**AACTCAGGTCGCTCTTCAGC**	**GCTTGATAGCCTCCAGCAAC**
Sqstm1	**NM_009360**	**AGCTTGTAAATGAGGCTTGGA**	**AGATGTCTCCGGATCGTTTC**
Stk11	**NM_011492**	**CACAAGCTGGATCACATTCC**	**GCGGTCAAGATCCTCAAGAA**
Tfam	**NM_009469**	**TAGTCAGCCAGGTCTCCACC**	**CTGCTGGGAAAGGAAATCAA**
Tgfβ1	**NM_011577**	**AAGTTGGCATGGTAGCCCTT**	**GCCCTGGATACCAACTATTGC**
Timm23	**NM_016897.3**	**AAGTAGCAACAAATCCACCAGC**	**AAATCAGCGTTCGAGTAACCC**
Timm44	**NM_011592.2**	**CTAGGCAGCGGAATCCAATTT**	**AGCAAGCCTGACAAAAACCCT**
Tnfα	**NM_013693.2**	**CAC AAG ATG CTG GGA CAG TGA**	**TCC TTG ATG GTG GTG CAT GA**
Tomm20	**NM_024214.2**	**GCCCTCTTCATCGGGTACTG**	**ACCAAGCTGTATCTCTTCAAGGA**
Tomm6	**NM_001164729.1**	**AGGTTCCAGACAACGTGGGA**	**AATGTCACTCAAGTTCCTGGC**

**Gene Name**	**Forward (5'->3')**	**Score**	**E-value**	**Reverse (5'->3')**
Col22a1	GGGGAACCTGGATACGCTAAA	42.1	0.003	CAAAGTACGCACACTGGGAG
Col24a1	TGCCTATATTGAGTCACCTCTCG	46.1	3.00E-04	TCTGTTGTTATTCCGAATGCTGA
Fkbp5	TCCACTACAAAGGGATGTTGTCA	46.1	3.00E-04	CGAGCCATAAGCATATTCTGGTT
Ppp1r3c	GCTCATTCACCACCTCTGAAG	42.1	0.003	GCTTGACACTGAGACATGGTT
Enox2	TGCAACAGCATCTGCTCAAAG	42.1	0.003	TGCACACAGTCTAGAACCACA
Ankrd1	GGAACAACGGAAAAGCGAGAA	42.1	0.003	GAAACCTCGGCACATCCACA
Sbk2	GAAGACAAGCCAATGGAAGTGT	44.1	8.00E-04	CCACCTCAGTTTGGACCAGG
Npr3	GGCGGCTCAAAAGATCGAG	38.2	0.021	CCATTGCCCTCCACGCTAC
Tomm5	ATGAAGCGGAAGATGCGTGAG	42.1	0.003	AGGGCCACGTAGATGAGGAA
Tomm7	ACCGTCGGTTTTAAGCCTACTT	44.1	8.00E-04	CCACTCTGCCACAGAATCGT
Tomm22	AGACCCTGTCGGAGAGACTC	40.1	0.008	TCTGAGCCACGAAGAGCGA
Tomm40	CCAGAGCATCACACCGTGTC	40.1	0.008	CCAGCGTTACTGTAGCCAACC
Tomm70a	AGCAAGCTATTCAGTGCTACAC	44.1	8.00E-04	AGGGCTTTCACATATTTGGGATT
Timm17a	TCCGAGGAAGTTTGACAGCTA	42.1	0.003	TAAGGCACCGCTAGTGATGGA
SH3BP5 (Sab)	GCAAAGTGTTCGAGGCTAATGA	44.1	8.00E-04	GAAAAGACCTCCTAGCAAGTCAG
